# Multi-Location Evaluation of Global Wheat Lines Reveal Multiple QTL for Adult Plant Resistance to Septoria Nodorum Blotch (SNB) Detected in Specific Environments and in Response to Different Isolates

**DOI:** 10.3389/fpls.2020.00771

**Published:** 2020-06-10

**Authors:** Michael G. Francki, Esther Walker, Christopher J. McMullan, W. George Morris

**Affiliations:** ^1^Department of Primary Industries and Regional Development, South Perth, WA, Australia; ^2^State Agricultural Biotechnology Centre, Murdoch University, Murdoch, WA, Australia

**Keywords:** Septoria nodorum blotch, QTL, genotype, isolate, environment, gene, interactions

## Abstract

The slow rate of genetic gain for improving resistance to Septoria nodorum blotch (SNB) is due to the inherent complex interactions between host, isolates, and environments. Breeding for improved SNB resistance requires evaluation and selection of wheat genotypes consistently expressing low SNB response in different target production environments. The study focused on evaluating 232 genotypes from global origins for resistance to SNB in the flag leaf expressed in different Western Australian environments. The aim was to identify resistant donor germplasm against historical and contemporary pathogen isolates and enhance our knowledge of the genetic basis of genotype-by-environment interactions for SNB response. Australian wheat varieties, inbred lines from Centro Internacional de Mejoramiento de Maiz y Trigo (CIMMYT), and International Center for Agricultural Research in the Dry Areas (ICARDA), and landraces from discrete regions of the world showed low to moderate phenotypic correlation for disease response amongst genotypes when evaluated with historical and contemporary isolates at two locations across 3 years in Western Australia (WA). Significant (*P* < 0.001) genotype-by-environment interactions were detected regardless of same or different isolates used as an inoculum source. Joint regression analysis identified 19 genotypes that consistently expressed low disease severity under infection with different isolates in multi-locations. The CIMMYT inbred lines, 30ZJN09 and ZJN12 Qno25, were particularly pertinent as they had low SNB response and highest trait stability at two locations across 3 years. Genome wide association studies detected 20 QTL associated with SNB resistance on chromosomes 1A, 1B, 4B, 5A, 5B, 6A, 7A, 7B, and 7D. QTL on chromosomes 1B and 5B were previously reported in similar genomic regions. Multiple QTL were identified on 1B, 5B, 6A, and 5A and detected in response to SNB infection against different isolates and specific environments. Known SnTox-*Snn* interactions were either not evident or variable across WA environments and SNB response may involve other multiple complex biological mechanisms.

## Introduction

Septoria nodorum blotch (SNB) caused by the fungal pathogen *Parastagonospora* (syn. ana, *Stagonospora*; teleo, *Phaeosphaeria*) *nodorum* (Berk.) Quaedvlieg, Verkley, and Crous is a significant necrotrophic disease on the leaf and glume of bread wheat. Disease epidemics cause significant yield losses in wheat growing regions of the world, including annual yield losses of 12.9% in Western Australia (WA; Murray and Brennan, 2009). SNB resistant varieties through breeding efforts are estimated to contribute 30% of pathogen control whereas cultural practices and fungicide application contributes the remaining 70% in WA (Murray and Brennan, 2009). Therefore, deploying new improved SNB resistant varieties whilst integrating on-farm management practices as a dual approach provide significant benefits to control the pathogen and disease.

*Parastagonospora nodorum* have asexual pycnidiospores and sexual ascospores where the latter is the primary source of inoculum (McDonald et al., 1994). There is a high degree of genetic diversity within and between isolate populations from major wheat growing continents with annual cycles of sexual reproduction contributing to population structure (Stukenbrock et al., 2006). Allele assortment, therefore, significantly influences isolate aggressiveness on the host plant in specific environments (Engle et al., 2006; Ali and Adhikari, 2008) whereby breeding for adult plant resistance (APR) would benefit from selection via natural infection or using a mixture of the most genetically diverse isolates for artificial inoculation in a particular environment (Cowger and Silva-Rojas, 2006). It is unlikely that any single isolate or a specific environment would provide sufficiently robust evaluation for host APR in the field as pathogen aggressiveness amongst isolates and host responses vary between wheat genotypes (Scharen and Eyal, 1983; Scharen et al., 1985; Eyal, 1999; Ali and Adhikari, 2008). Consequently, selection of isolates from diverse geographic origins and their effect on wheat cultivars in different environments are important considerations when evaluating disease symptoms and understanding the genetic control of resistance to SNB. Moreover, variation in heading date and height are important determinants in breeding and selection whereby wheat lines ranked as SNB resistant variation could be misclassified particularly for late maturing genotypes that escaped disease infection (Francki, 2013). In some cases, genetic factors controlling height and heading date were, indeed, linked to genes controlling SNB response rather than pleiotropic effects of agronomic characteristics affecting disease evaluation (Shankar et al., 2008; Francki et al., 2011).

Genetic analysis has shown seedling resistance to be under the control of minor genes and independent to APR genes when a bi-parental mapping population was evaluated for SNB response specifically in WA environments (Shankar et al., 2008) with general disease susceptibility often increasing toward physiological maturity of the plant (Develey-Rivière and Galiana, 2007). Infection at or beyond ear emergence is the most damaging, reducing photosynthetic capacity and grain yield (Mullaney et al., 1983; Spadafora et al., 1987; Wainshilbaum and Lipps, 1991). Given disease symptoms proliferate in the warmer spring temperatures in WA as the crop matures, APR is therefore, a high priority in breeding to maintain photosynthetic capability in the leaf and glume when SNB epidemics are at the greatest in-season risk. APR is under polygenic control and genes for leaf and glume resistance have previously been reported to be minor with additive effects (Rosielle and Brown, 1980; Fried and Meister, 1987; Bostwick et al., 1993). A number of genetic studies based on bi-parental mapping populations identified quantitative trait loci (QTL) specifically for SNB resistance on the flag leaf and glume on chromosomes 1B, 3B, 2A, 2D, 4B, and 5B using either mixed isolates as inoculum or natural infection in field environments (reviewed in Francki, 2013). In some instances, QTL for SNB resistance were not detected in all environments (Shankar et al., 2008; Francki et al., 2011; Lu and Lillemo, 2014) indicating that host resistance is controlled by genes expressed in response to different isolates, environments or isolate-by-environment interactions. Moreover, a number of the QTL identified using moderate density molecular marker maps appeared to be co-located on the same regions, such as those on chromosomes 1B, 2A, 2D, and 5B and genes at these loci controlled SNB resistance for different isolates and environments (Shankar et al., 2008; Francki et al., 2011). However, higher resolution genetic maps using the iSelect Infinium 90K single nucleotide polymorphic (SNP) genotyping array (Wang et al., 2014) allowed for and enabled the discrimination of co-locating QTL for SNB resistance into discrete but linked loci (Francki et al., 2018). Multiple loci on the same chromosome, therefore, responded independently to different isolates and environments.

Although genetic maps with high density SNP markers have been beneficial in discriminating genetic loci in QTL mapping, trait variation in bi-parental mapping populations are constrained to alleles from either parent. Genome wide association studies (GWAS) enhances resolution in marker-trait associations whilst simultaneously evaluating a broader gene pool and alleles for desired trait variation. Alleles and marker polymorphisms in a GWAS panel are associated with traits of interest through historical recombination events and linkage disequilibrium (LD). There are increasing reports on the application of GWAS for genetic analysis of traits, including disease resistance. For example, marker-trait associations linked to resistance against stem and stripe rust (Bajgain et al., 2015; Bulli et al., 2016; Gao et al., 2016; Maccaferri et al., 2016) and Fusarium head blight (Arruda et al., 2016) by GWAS further unraveled the genetic complexity of fungal resistance and identified known and discovered novel loci with an expanded repertoire of molecular markers linked to genes controlling disease resistance. Therefore, the application of GWAS allows the evaluation of wider accessions for disease response whilst simultaneously identifying new marker-trait associations with finer mapping resolution based on LD and discovery of new alleles not necessarily represented in parents of bi-parental mapping populations. There are reports for identifying the genetic control of SNB in wheat using GWAS. Included are those for analyzing seedling resistance in a wheat panel of 528 lines that were screened using a single *P. nodorum* isolate in a single controlled environment using lower density DArT and the Illumina iSelect beadchip 9K SNP assay (Adhikari et al., 2011; Gurung et al., 2014). Considering plant response to disease changes through physiological development from seedling to maturity (Develey-Rivière and Galiana, 2007) and environmental influences on fungal isolate aggressiveness (Pariaud et al., 2009), GWAS analysis using the iSelect Infinium 90K genotyping array (Wang et al., 2014) provides opportunities for a detailed investigation on discrete QTL controlling adult plant response to SNB in multi-environments based on evaluation of a wider gene pool. In recent GWAS studies a number of QTL were detected in only one field environment with some detected in two or more environments and only one QTL on chromosome 2D and 2A detected in all environments (Ruud et al., 2019; Lin et al., 2020). It appears, therefore, that host response to SNB is largely variable and complex where several loci are detected in specific environments indicating considerable genotype-environment interactions.

*Parastagonospora nodorum* produces a range of necrotrophic effector (NE) proteins that induce necrosis in wheat that harbor corresponding sensitivity genes (McDonald and Solomon, 2018). Several NE-host interactions have been identified and mapped in wheat including SnToxA-*Tsn1* interaction on chromosome 5B (Liu et al., 2006; Friesen et al., 2009), SnTox1-*Snn1* on 1B (Phan et al., 2016); SnTox2-*Snn2* on 2D (Friesen et al., 2009), SnTox3-*Snn3-B1* on 5B (Friesen et al., 2008; Ruud et al., 2017; Downie et al., 2018), SnTox4-*Snn4* on 1A (Abeysekara et al., 2009), SnTox5-*Snn5* on 4B (Friesen et al., 2012), SnTox6-*Snn6* on 6A (Gao et al., 2015), and SnTox7-*Snn7* on 2D (Shi et al., 2015). Although these studies provided a basis to determine biological processes involved in SNB interaction with wheat, the effect of *Snn* loci contributing to SNB response against WA isolates in different field environments is not well defined.

The aim of this study was to evaluate SNB response of flag leaf in 232 diverse bread wheat accessions from global breeding programs and discover the genetic interaction of host resistance to different *P. nodorum* isolates in SNB trials by GWAS using the iSelect Infinium 90K genotyping array. The evaluation for SNB response was done in two locations annually for 3 years by inoculating field trials with a mixture of historical and contemporary *P. nodorum* isolates collected from diverse geographical regions in WA and the potential role of NE-host interactions elucidated by comparing QTL for SNB response with known *Tsn* and *Snn* loci. The outcome of the study will refine our knowledge on host genes responding to SNB and the interplay between isolate diversity and different environments to improve breeding resistance in bread wheat.

## Materials and Methods

### Plant Material

The GWAS accession panel consisted of 71 wheat lines from Australian origin, 72 inbred and commercial lines from Centro Internacional de Mejoramiento de Maiz y Trigo (CIMMYT), 78 inbred lines from International Center for Agricultural Research in the Dry Areas (ICARDA), and 11 landraces from various origins. Inbred lines were accessed through the CIMMYT Australia ICARDA Germplasm Evaluation (CAIGE) project^1^. Pedigrees and origins of 232 wheat accessions are provided in Supplementary Table S1.

### Isolates and Preparation of Inoculum

*Parastagonospora nodorum* isolates from various wheat growing regions of WA were sourced from the culture collection at the Department of Primary Industries and Regional Development (DPIRD), formerly the Department of Agriculture and Food WA. Inoculum used for evaluation of SNB in field trials each year consisted of a mixture of 16–20 isolates where at least 35% of the inoculum represented contemporary isolates collected from the previous year of the trials. Mixed inoculum used in each year included at least four of the same isolates represented in successive years. Supplementary Table S2 provides details of the isolates used in inoculum each year, including the origin and year of collection. Fungal cultures were prepared by growing the isolates on sterile wheat grain for 3 months to induce formation of pycnidia and pycnidiospores (Fried, 1989). Fungal cultures were air-dried and ground to a coarse powder, equal amounts of each isolate was mixed and stored at 4°C. The mixed fungal isolates were rehydrated to a suspension of 10^6^ spores/ml with 0.5% gelatine prior to use as inoculum in field trials.

### Field Trials

Trials in 2016 and 2017 were sown at Northam and Katanning, and Northam and Manjimup in 2018. Locations were selected based on varying mean annual temperatures, rainfall and maximum relative humidity (Northam: 18.1°C, 432 mm, 71%; Katanning: 15.4°C, 470 mm, 70%; and Manjimup: 14.7°C, 981 mm, 76%). Trials were arranged in completely randomized block design with 3 replications for each accession with 0.6 m spacing between plots. Each plot consisted of a single 1.9 m length row with 0.4 m row spacing in 2016 and 2017 trials and two rows of 1.9 m length with 0.2 m row spacing in 2018. A spreader 2-row plot of the susceptible early maturing variety “Amery” was adjacent to each treatment plot. Susceptible check cultivars (6 reps) comprising cultivars “Amery” (early maturing), “Arrino” (early-mid maturity), “Millewa” (mid-late maturity), “EGA2248” (mid-late maturity), and “Scout” (late maturity) were included in all trials. Trials were rainfed or irrigated with above ground sprinklers to simulate a rainfall event and induce disease when required.

Inoculum (10^6^ spores/ml with 0.5% gelatine) was applied using a motorized mister commencing at Feekes growth stage 3–4 with a further three inoculations at 10–14 day intervals. Inoculum was applied at a rate of 28.5 m^2^/L prior to a rainfall event. Sprinkler irrigation was applied to trials in the absence of rainfall within 2 days post inoculation. SNB response on the flag leaf was visually assessed using percent leaf area diseased (PLAD) scale on susceptible check cultivars where 0% and 100% were scored as highly resistant and highly susceptible, respectively. All accessions were scored in each trial when at least 2 susceptible check varieties had PLAD scores >70%. Disease ratings were scored for 5 random individual plants from the middle of each plot and mean plot scores were used in statistical analysis.

Measurements for plant height (cm) were taken as the distance from the top of the soil to the top of the heads (excluding awns). Three measurements were taken in each plot and mean plot scores used for analysis. Heading date was measured as days from date of sowing to when 50% of the ears were fully emerged for each plot.

### Analysis of SNB Disease Response

All statistical analyses for phenotypic evaluation were done using Genstat, 19th edition^2^. Disease and agronomic data were tested for normality and error variance homogeneity across environments. Residuals plotted against fitted values revealed a random distribution (data not shown) indicating there was no need for data transformation. Generalized linear models and linear mixed models were used in phenotypic analysis of trait data. Treatment factors and co-variates were fitted to fixed models to estimate main effects and interactions. Finlay-Wilkinson joint regression analysis was used to compare genotypes for SNB response and agronomic traits at two locations across 3 years. Broad-sense heritability estimates were calculated using the formula *H*^2^ = $\sigma_{\text{g}}^{2}$/$\sigma_{\text{g}}^{2}$ + $\sigma_{\text{e}}^{2}$/*r*, where $\sigma_{\text{g}}^{2}$, and $\sigma_{\text{e}}^{2}$ are the genotypic and error variance, respectively, and *r* is the number of replications.

### Genotyping

DNA samples from wheat accessions were assayed using the 90K Infinium SNP chip array. Raw intensity data was analyzed in GenomeStudio, and NormTheta and NormR values for each SNP were extracted. A custom perl script was used to cluster samples and assign genotypes to known polymorphisms. Homozygous genotypes were called when a sample was located at a previously identified cluster from a bi-parental mapping population, whether they had been genetically mapped or not. Clusters that had been genetically mapped in bi-parental mapping populations were assigned to chromosomes and clusters reported in the wheat SNP Consensus map 90K Array (Wang et al., 2014) were also assigned a map position, with some SNP having multiple loci. Physical location of genetic markers were extracted from the International Wheat Genome Sequencing Consortium (IWGSC) RefSeq v1.0 physical map via https://wheat-urgi.versailles.inra.fr/ (Alaux et al., 2018).

The *Tsn1* locus was genotyped across lines of the GWAS panel using the linked marker *fcp620* as previously described (Zhang et al., 2009). The effect of sensitive versus insensitive alleles was then determined for each environment (*p*-value threshold *p* < 0.05) to establish whether *Tsn1* had a significant effect on PLAD scores. The genotypes for *fcp620* were also compared to the marker-trait associations (MTA) co-locating with *Tsn1*, *IWB14942*, and *IWB43679*.

### Population Structure, Genome-Wide Association and Statistical Analysis

Monomorphic markers and markers with less than 80% call rate were removed from the dataset. Markers with minor allele frequencies (MAF) less than 5% were removed prior to analysis, reducing the total number of SNPs to 20,563. The data was then split into three separate analyses, one set containing the filtered data set of 20,563 SNPs, a set where the markers were further filtered to include SNPs with no less than 90% call rate and pruning the data based on a LD of *r*^2^ ≤ 0.2 using the PLINK 1.9 command “–indep-pairwise 50 2 0.2,” reducing the data set to 2,941 SNPs, and a third set where the markers were filtered to include SNPs with no less than 90% call rate and pruning the data based on a LD of *r*^2^ ≤ 0.1 using the PLINK 1.9 command “–indep-pairwise 50 2 0.1,” reducing the data set to 1,142 SNPs.

To generate a Q matrix the LD pruned data (*r*^2^ ≤ 0.1) was imported into STRUCTURE version 2.3.4. (Pritchard et al., 2000; Falush et al., 2003, 2007; Hubisz et al., 2009) and applied to full set, pruned *r*^2^ ≤ 0.2 and pruned *r*^2^ ≤ 0.1 marker datasets for MTA. A burn-in/MCMC of 20,000/40,000 was applied, selecting the Admixture model with correlated allele frequencies from *K* = 1–10 with 10 independent runs each. The output was imported into Structure Harvester (Earl and von Holdt, 2012) and determined that *K* = 2. CLUMPP version 1.1.2 (Jakobsson and Rosenberg, 2007) was implemented to summarize the output as a Q matrix for use in association analysis in TASSEL v.5.2.52 (Bradbury et al., 2007). Within TASSEL a genotypic kinship matrix (K) was estimated by selecting the “Centered_IBS” method. General linear model (GLM; Q), GLM (PCA), mixed linear model (MLM; Q + K), and MLM (PCA + K) were all initially explored with visual assessment of quantile-quantile plots (Q-Q plots). The suitable number of PCs for each trait was determined by testing one through 15 PCs. The option “P3D” was not selected during the MLM analysis with the variance component re-estimated after each marker. After assessing Q-Q plots it was determined that MLM (PCA + K) was the most suitable method, accounting for both population structure and cryptic relatedness. Significant associations were only detected in the full data set with no significant associations detected in either pruned data sets. The pruned data sets were removed from further analysis. The R programs “qqman” and “Rcolorbrewer” were used to draw Manhattan plots (Turner, 2017; R Core Team, 2018). Two-dimensional displays of the top PCs were drawn in R.

A genome-wide significance threshold for MTAs was set at *p* < 2.43 × 10^–6^ [−log_10_ (*p*) > 5.61] using Bonferroni correction with α = 0.05. Bonferroni correction is highly conservative and reduces type I errors, however, is most practical when all tests are independent (Bland and Altman, 1995; Abdi, 2007). To estimate the number of independent tests the tagger function in Haploview was implemented as described in Maccaferri et al. (2016) with a *r*^2^ of 0.1. This returned a genome-wide threshold significance of *p* < 7.65 × 10^–5^ [−log_10_ (*p*) > 4.12] and was considered a moderate level of significance compared to the Bonferroni corrected threshold. As reported in a number of related studies a threshold of significance of *p* < 1 × 10^–3^ [−log_10_ (*p*) > 3.00] was included as a suggestive level of significance (Maccaferri et al., 2015; Alomari et al., 2017; Muqaddasi et al., 2019).

Linkage disequilibrium was assessed to identify MTAs that occurred as single entities or clusters of SNPs in strong LD. The “–block” function in PLINK 1.9 was utilized to identify intra-chromosomal SNPs in strong LD as previously defined (Gabriel et al., 2002) with the bottom of the 90% D-prime confidence interval greater than 0.70 and the top of the confidence interval at least 0.98 (Purcell et al., 2007). LD decay was estimated to gage the approximate size of QTL intervals. Marker pairwise *r*^2^ values were calculated in PLINK 1.9 with a sliding window of 50 and then LD decay curves fitted by non-linear regression for each subgenome (A, B, and D) as previously described (Marroni et al., 2011) with decay of *r*^2^ against distance. LD decay plots were drawn in R with a critical threshold of *r*^2^ = 0.2 which represented the LD half decay point (R Core Team, 2018). QTL were defined as having moderate to highly significant MTA for a single SNP locus. Linkage decay values were used as estimates for a QTL interval when multiple moderate to highly significant MTA were identified within a similar genomic region.

The most significant SNP marker was chosen as a representative for all QTL and their individual and collective effect of allele stacking on PLAD scores for each environment was assessed and *P*-values calculated in R (R Core Team, 2018).

## Results

### Phenotypic Analysis of SNB Response

The population mean values for SNB response measured as PLAD ranged from 25.0 to 53.0 (Table 1) indicating variable disease pressure across environments and years. Analysis of variance indicated significant differences between genotypes for PLAD, heading date and height in each environment (Table 1). High broad sense heritability was observed for PLAD in each of the two locations across three years (*H^2^* = 0.64–0.88) and similarly for heading date and plant height (Table 1).

**TABLE 1:** Summary statistics for SNB response. Trait measurements include percent leaf area diseased (PLAD) heading date (HD) and plant height (HGT) from two locations annually in years 2016–2018.

	2016						2017						2018					
			
	Northam			Katanning			Northam			Katanning			Northam			Manjimup		
						
	PLAD	HD	HGT	PLAD	HD	HGT	PLAD	HD	HGT	PLAD	HD	HGT	PLAD	HD	HGT	PLAD	HD	HGT
Minimum	5.0	91.0	69.0	20.0	92.0	64.0	3.0	85.0	67.0	3.0	103.0	69.0	3.0	91.0	77.0	2.0	86.0	73.0
Maximum	92.0	129.0	110.0	90.0	139.0	102.0	85.0	116.0	106.0	85.0	132.0	101.0	75.0	122.0	143.0	97.0	133.0	123.0
Grand Mean	44.1	108.0	85.7	53.0	125.9	82.2	39.0	101.4	80.0	30.0	120.1	84.0	25.0	104.8	105.0	38.0	111.7	94.0
Median	45.0	108.0	86.0	53.0	127.0	82.0	38.0	102.0	80.0	27.0	121.0	84.0	22.0	104.0	106.0	36.0	110.0	94.0
Mode	45.0	109.0	83.0	40.0	131.0	85.0	33.0	102.0	79.0	18.0	120.0	86.0	28.0	99.0	109.0	53.0	115.0	94.0
ANOVA (*P*)	<0.001	<0.001	<0.001	<0.001	<0.001	<0.001	<0.001	<0.001	<0.001	<0.001	<0.001	<0.001	<0.001	<0.001	<0.001	<0.001	<0.001	<0.001
LSD (*P* < 0.05)	23.0	4.5	7.0	23.0	9.2	10.6	26.6	6.5	10.1	32.3	8.4	13.2	17.9	5.1	8.1	19.6	4.9	6.8
CV (%)	33.1	2.6	5.0	26.8	4.5	8.0	43.0	4.1	7.8	47.1	3.1	6.9	45.1	3.0	4.8	30.6	2.8	4.5
*H*^2^	0.76	0.92	0.85	0.67	0.69	0.65	0.76	0.83	0.63	0.64	0.81	0.73	0.74	0.48	0.47	0.88	0.94	0.90

Pearson’s correlation co-efficient for PLAD scores within and between years was low to moderate but highly significant (*r* = 0.272 to 0.568, *P* < 0.001; Table 2). It appears, therefore, that SNB response of many individual genotypes is variable even when inoculated with the same isolates across different locations in the same year indicating host-by-isolate-by-environment interactions. The exception was between Northam and Manjimup in 2018 where correlation for PLAD was high (*r* = 0.797, *P* < 0.001; Table 2) indicating that, in some instances, the relative response of genotypes to SNB between environments in the same year was similar when inoculated with the same isolates.

**TABLE 2:** Pearson’s correlation co-efficient of SNB response across locations within and between years 2016–2018.

	**Northam 2016**	**Katanning 2016**	**Northam 2017**	**Katanning 2017**	**Northam 2018**	**Manjimup 2018**
Northam 2016	–					
Katanning 2016	0.568***	–				
Northam 2017	0.272***	0.328***	–			
Katanning 2017	0.455***	0.455***	0.431***	–		
Northam 2018	0.339***	0.387***	0.338***	0.494***	–	
Manjimup 2018	0.460***	0.433***	0.385***	0.513***	0.797***	–

***P < 0.001.

The contribution of genotype, environment and their interactions in the same year was further analyzed by fitting genotype, environment and their interactions as terms in linear mixed models. Among the three sources of variation, the largest proportion of SNB response was significantly (*P* < 0.01) accounted by genotypes (72.94–79.71%) followed by genotype-by-environment interactions (10.22–22.63%) and environment (4.43–10.07%) for each year (Table 3). Moreover, a highly significant (*P* < 0.001) but smaller proportion of variation was accounted by genotypes at two locations across 3 years (44.52%) whereby genotype-by-environment interactions (34.52%), and environment (20.96%) was higher compared to their respective sources of variation across environments within years (Table 3). The genotype-by-environment interactions within and between years indicated that either varying host response to disease, genetically distinct isolates, diverse isolate virulence, or aggressiveness or a combination of these factors interact to significantly influence the expression of SNB response across genotypes in different environments.

**TABLE 3:** Linear mixed model analysis for genotypes, environments and their interactions for 232 wheat lines responding to SNB within years and across all environments in 2016–2018.

	2016			2017			2018			All environments (2016–2018)		

Source of variation	Wald statistic	*P*^a^	%Var^b^	Wald statistic	*P*	%Var	Wald statistic	*P*	%Var	Wald statistic	*P*	%Var
Genotype (G)	1238.7	<0.001	76.49	1166.3	<0.001	72.94	3051.6	<0.001	79.71	3364.1	<0.001	44.52
Environment (E)	104.7	<0.001	6.47	70.9	<0.001	4.43	385.4	<0.001	10.07	1584.0	<0.001	20.96
GxE	276.0	<0.01	17.04	362.3	<0.001	22.63	391.1	<0.001	10.22	2608.8	<0.001	34.52

*^*a*^*F*-test probability of Wald statistic. ^*b*^Percentage of variation associated with each term or interaction.*

### Genotype Performance Using Joint Regression Analysis

Despite highly significant genotype-by-environmental interactions for PLAD within and between years (Table 3), means of each genotype were fitted with average environmental means using a Finlay and Wilkinson joint regression model to identify genotypes with low mean PLAD scores at two locations across 3 years and ranked in ascending order based on sensitivity to SNB response (Table 4). Genotypes with PLAD scores <30.0 and with similar heading date and plant height to the susceptible check varieties were determined as consistently expressing SNB resistance. A total of 19 genotypes had mean PLAD values across all locations ranging from 15.33 to 28.94 compared to control susceptible varieties with similar heading dates and plant height (Table 4). The CIMMYT inbred lines ZJN12 Qno 25 and 30ZJN09, in particular, were identified as having low SNB severity with high stability and lowest mean square deviation indicating greater predictable SNB disease response in any given environment (Table 4). Interestingly, ZJN12 Qno 25, and 30ZJN09 had comparable or improved resistance to SNB but with higher stability and predictability than other resistant lines, 6HRWSN125, and EGA Blanco (Table 4) that have been deployed as donor parents in doubled haploid populations for QTL studies (Shankar et al., 2008; Francki et al., 2011). Moreover, ZJN12 Qno 25, 30ZJN09, 6HRWSN125, and EGA Blanco have distinctly different pedigrees (Supplementary Table S1) so it is likely that the source of SNB resistance is from different parental origins.

**TABLE 4:** Summary of resistant wheat genotypes relative to susceptible controls and their mean PLAD scores, heading date and plant height at two locations across 3 years (2016–2018) using joint regression analysis.

PLAD				Heading date	Plant height
		
Varieties/Inbreds	Mean (s.e.)	Sensitivity (s.e.)^a^	Mean square deviation^b^	Mean (s.e.)	Mean (s.e.)
**Resistant**					
4:ZIZ13	24.68 (4.05)	−0.211 (0.43)	488.9	110.3 (1.07)	85.35 (1.53)
Tammin	24.39 (3.93)	0.158 (0.43)	156.7	113.2 (1.04)	84.30 (1.41)
ZJN12 Qno 25	15.33 (3.91)	0.587 (0.39)	74.1	111.1 (1.04)	95.74 (1.37)
Envoy	28.94 (3.80)	0.682 (0.39)	178.5	114.4 (1.04)	82.41 (1.37)
Ajana	24.75 (3.80)	0.748 (0.39)	151.4	111.8 (1.04)	86.48 (1.37)
30ZJN09	23.57 (3.93)	0.838 (0.43)	93.5	113.7 (1.04)	91.66 (1.41)
Gladius	22.50 (3.80)	0.859 (0.39)	189.6	114.6 (1.04)	82.69 (1.37)
3:ZIZ12	27.87 (5.45)	0.957 (0.59)	161.2	108.3 (2.14)	86.24 (1.96)
WAWHT2046	24.05 (4.05)	1.048 (0.43)	112.3	104.4 (1.04)	93.22 (1.41)
ZWW10 Qno 127	24.03 (3.91)	1.223 (0.39)	116.5	114.0 (1.07)	90.12 (1.41)
ZVS07 Qno 227	26.12 (3.93)	1.238 (0.43)	350.0	112.9 (1.04)	97.34 (1.41)
52:ZIZ12	22.96 (5.79)	1.321 (0.52)	180.4	113.7 (4.33)	90.02 (2.14)
Mace	26.69 (4.05)	1.329 (0.43)	194.6	114.8 (1.07)	86.28 (1.46)
6HRWSN125	28.17 (3.80)	1.414 (0.39)	218.1	112.7 (1.07)	91.76 (1.37)
EGA Castle Rock	22.26 (4.03)	1.437 (0.40)	141.5	111.8 (1.11)	94.15 (1.41)
ZVS09 Qno 133	25.13 (3.93)	1.478 (0.43)	187.0	113.8 (1.04)	89.67 (1.41)
ZWW09 Qno 125	25.71 (3.91)	1.501 (0.39)	147.0	112.9 (1.07)	97.55 (1.41)
EGA Blanco	21.07 (3.80)	1.544 (0.39)	169.6	114.9 (1.04)	87.50 (1.37)
ZEE10 Qno 77	21.36 (3.80)	1.673 (0.39)	200.7	114.3 (1.07)	96.29 (1.41)
**Susceptible**					
Amery	67.89 (3.80)	0.338 (0.39)	144.9	101.4 (1.04)	86.20 (1.37)
Millewa	69.22 (3.80)	0.826 (0.39)	356.8	111.4 (1.04)	86.40 (1.41)
Arrino	60.33 (3.80)	0.871 (0.39)	222.8	105.3 (1.04)	83.52 (1.37)
EGA 2248	58.64 (3.91)	1.231 (0.39)	222.5	110.4 (1.04)	91.11 (1.37)
Scout	46.33 (3.82)	1.292 (0.39)	183.3	114.4 (1.04)	89.63 (1.37)

*^*a*^Genotypes ranked in ascending order from the most stable assessed by sensitivity of PLAD response to environmental effects. ^*b*^Predictability of response to SNB assessed by mean square deviation.*

### Fixed Effects of Morphological Traits on SNB Response

Pleiotropic effects of morphological characteristics can have significant implications when interpreting the genetic control of SNB response and often exacerbated in QTL studies when populations have significant differences in trait measurements. There was a significant and moderate to high negative correlation between heading date and PLAD scores (*r* = -0.51 to -0.86, *P* < 0.001) with similar negative correlation between plant height and PLAD scores (*r* = -0.52 to -0.79, *P* < 0.001) in all environments supporting potential pleiotropic effects of morphological characteristics on disease evaluation. Since heading date and plant height were significantly different (*P* < 0.001) between genotypes in each environment (Table 1), they were fitted as variates in a linear mixed model to estimate the significance of any main fixed effects and their interactions on PLAD. Heading date had highly significant (*P* < 0.001) main effects on SNB response at two locations across 3 years when adding to or sequentially dropping terms from the fixed model whereas plant height had significant main effects (*P* < 0.05) in most environments (Table 5). There was no significant heading date-by-height interactions (*P* > 0.05) in any of the environments (Table 5). Heading date and plant height were, therefore, fitted as co-variates in general linear model and adjusted mean PLAD scores were used for subsequent genetic analysis to reduce spurious MTA with agronomic characteristics and improve the accuracy of association of SNP markers with genes controlling PLAD response in GWAS.

**TABLE 5:** Results of fixed effect model to estimate the significance of main effects of heading date and plant height and their interactions on PLAD.

Fixed effects on PLAD response	Northam 2016			Katanning 2016		
		
	*F*	*P*	Estimate (SE)	*F*	*P*	Estimate (SE)
Heading date	248.10	*P* < 0.001	−1.707(0.11)	82.13	*P* < 0.001	−0.8440(0.09)
Height	5.36	*P* = 0.021	0.210 (0.09)	34.02	*P* < 0.001	0.5149 (0.09)
Heading Date.Height	2.07	*P* = 0.150	−0.022(0.01)	2.71	*P* = 0.100	0.0244 (0.01)

	**Northam 2017**			**Katanning 2017**		
		
	***F***	***Sig***	**Estimate (SE)**	***F***	***Sig***	**Estimate (SE)**

Heading date	208.40	*P* < 0.001	−1.902(0.13)	406.71	*P* < 0.001	−2.202(0.11)
Height	7.20	*P* = 0.008	−0.293(0.11)	0.05	*P* = 0.819	0.0183 (0.08)
Heading Date.Height	0.29	*P* = 0.591	0.010 (0.02)	0.60	*P* = 0.439	0.0144 (0.02)

	**Northam 2018**			**Manjimup 2018**		
		
	***F***	***Sig***	**Estimate (SE)**	***F***	***Sig***	**Estimate (SE)**

Heading date	219.16	*P* < 0.001	−1.298(0.09)	285.67	*P* < 0.001	−1.526(0.09)
Height	63.69	*P* < 0.001	−0.4824(0.06)	153.26	*P* < 0.001	−1.015(0.08)
Heading Date.Height	0.19	*P* = 0.665	0.0047 (0.01)	1.29	*P* = 0.257	−0.0140(0.01)

### Population Structure and Linkage Disequilibrium

**FIGURE 1:**
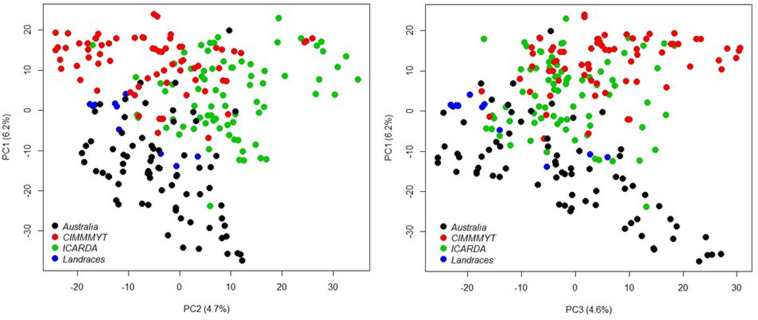
Principal component analysis of 232 wheat genotypes using 20,563 markers filtered from the 90K Infinium SNP chip array. Different colors represent genotype origins: black, Australian cultivars; red, CIMMYT inbred lines; and green, ICARDA inbred lines and blue, landraces.

**FIGURE 2:**
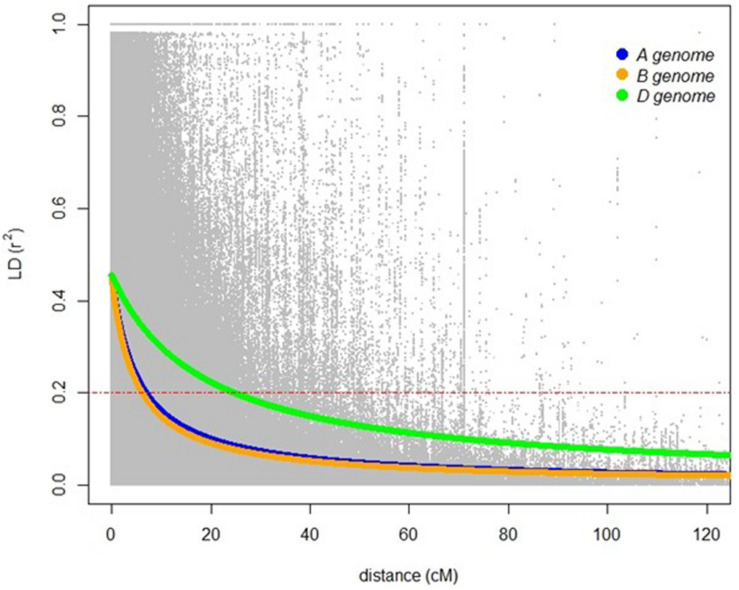
Linkage disequilibrium based on 20,563 SNP markers for the A (blue), B (orange), and D (green) genomes. Red dashed lines indicates LD decay level (*R*^2^ = 0.2).

A total of 20,563 markers filtered from the 90K Infinium SNP chip array was used to investigate the relatedness of the GWAS panel based on principal component analysis (PCA). PCA described 15.6% of the genetic variance between PC 1, 2, and 3 (6.2, 4.7, and 4.7%, respectively) and regarded as having low population structure (Figure 1). Despite wheat lines sourced from different breeding programs and continents and selected based on different pedigrees (Supplementary Table S1), demarcation for clustering into distinct sub-populations was not apparent (Figure 1) indicative of shared genetic relatedness of distant ancestors in inbred lines and varieties. A comparison between the entire 20,563 SNP marker dataset and the LD pruned datasets (2,491 SNP with no less than 90% call rate and LD of *r*^2^ ≤ 0.2 SNP, and 1,142 SNP with no less than 90% call rate and LD of *r*^2^ ≤ 0.1) showed no difference in principal component analysis and population structure, with the top PCs accounting for slightly less of the total variation in the LD pruned datasets (Figure 1 and Supplementary Figure S1). The extent of LD of the GWAS panel was estimated based on the pairwise squared correlation coefficients (*R*^2^) for the 20,563 SNP loci. LD decay was estimated by non-linear regression at 6.97 cM and 6.14 cM for the A and B subgenomes, respectively, whereas the D subgenome had a significantly higher LD of 24.62 cM for threshold *R*^2^ = 0.2 (Figure 2).

### Marker-Trait Associations for SNB Response

**FIGURE 3:**
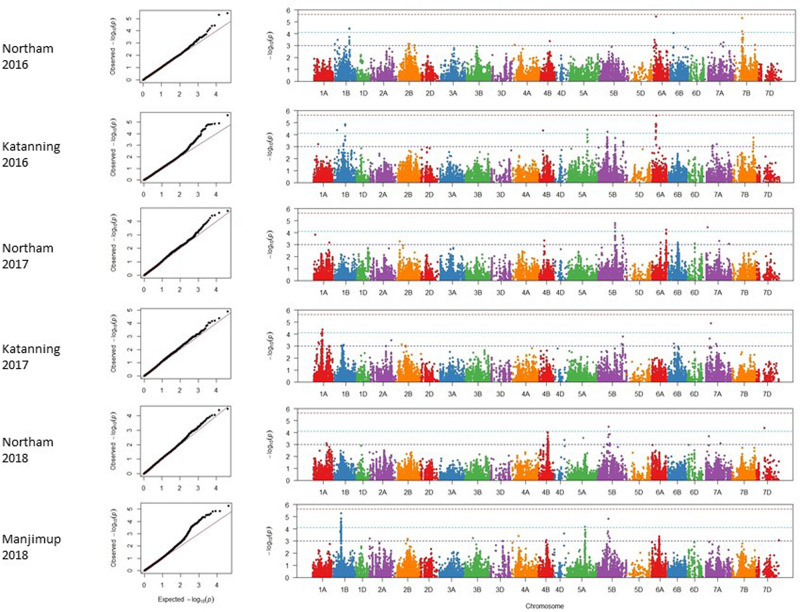
Genome wide association scans for adjusted mean PLAD scores for two locations across 3 years (2016–2018). Q-Q and Manhattan plots are shown to the left and right, respectively. Horizontal dotted lines in each environment represent suggestive (bottom), significant (middle), and highly significant (top) threshold levels for marker-trait associations.

A mixed linear model (MLM) was applied to account for any confounding effects of population structure and cryptic relatedness and reduce rate of false positives or spurious SNP marker associations linked to genes controlling SNB resistance. Adjusted mean PLAD values from six individual environments in 2016–2018 were used to reduce any confounding effects of heading date and height and analyzed using 20,563 filtered SNP markers. Analysis of quantile-quantile (Q-Q) plots for adjusted mean PLAD scores showed deviations of the observed association compared to the association statistics expected under the null hypothesis of no association (Figure 3), indicating SNP markers were associated with trait variation at two locations across 3 years. Association tests were analyzed independently for each of the environments with thresholds of *p* < 2.43 × 10^–6^ [−log_10_ (*p*) > 5.61], *p* < 7.65 × 10^–5^ [−log_10_ (*p*) > 4.12], and *p* < 1 × 10^–3^ [−log_10_ (*p*) > 3.00] ranking as a high, moderate and suggestive levels of significance, respectively, in Manhattan plots (Figure 3).

A total of 47 SNP loci associated with PLAD with moderate to high significance were detected on nine chromosomes at two locations across 3 years including 1A, 1B, 4B, 5A, 5B, 6A, 7A, 7B, and 7D (Table 6). None of the SNP markers detected for SNB were associated with known loci controlling variation for heading date and plant height (Supplementary Tables S3, S4 and Supplementary Figures S2, S3) using moderate to high threshold values of *p* < 7.65 × 10^–5^ [−log_10_(*p*) > 4.12] so it appears that markers are associated with disease response and not a pleiotropic effect of agronomic characteristics. The number of QTL detected for each environment was variable. A minimum of two QTL were detected for Katanning 2017 and Northam in 2018 with a maximum of seven QTL detected for Katanning in 2016 (Figure 3 and Table 6). The majority of MTA were detected on the A and B subgenomes with fewer genes controlling PLAD on the D subgenome when the GWAS panel was evaluated in all environments (Figure 3). Although SNP were evenly spaced across the genome, the average distance between SNP ranged from 0.52 cM in the full dataset to 9.06 cM in the smaller pruned dataset of 1,142 SNP. The full and pruned dataset had large gaps of 79.53 cM and 135.67 cM, respectively, both on the D subgenome. SNP per chromosome ranged from 83 SNP to 1907 SNP for the full dataset and 19 SNP to 111 SNP for the pruned dataset of 1,142 (Supplementary Table S5).

**TABLE 6:** Summary of SNP marker associations with PLAD scores from two locations across 3 years in 2016–2018. Gray shading represent SNP markers that are in strong LD as defined by Gabriel et al. (2002).

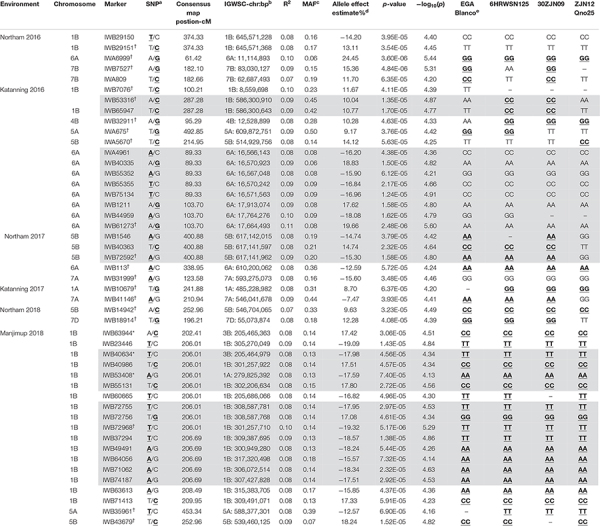

^*a*^Desirable SNP for reduced disease, based on the allele effect estimate, is in bold and underlined. ^*b*^IWGSC: IWGSC RefSeq v1.0, Chr: Chromosome, bp: base pairs. ^*c*^MAF: minor allele frequency. ^*d*^The effect estimates the difference between the average phenotypic values of the homozygous A genotype relative to the homozygous B genotype. ^*e*^Allele scores of each SNP marker for four wheat genotypes showing low PLAD scores at two locations across 3 years (Table 4). ^†^SNPs selected for analysis of allele stacking. *SNPs allocated to chromosome 1B on the genetic consensus map but having best Blastn hit on other chromosomes in the IWGSC RefSeq v1.0. -data not available.

Since genotype-by-environment interaction was significant (Table 3) and the disease correlation showed low to moderate Pearson’s coefficients between environments (Table 2), it was expected that the same QTL would not necessarily be detected across environments within and between years. A total of 20 QTL were identified at two locations across 3 years (Table 6). The locus at 539.46 Mbp to 546.70 Mbp on 5B was represented by *IWB14942* and *IWB43679* in Northam and Manjimup in 2018, respectively, but neither these SNP nor others at this locus were detected in the remaining four environments in 2016 and 2017 (Table 6) indicating that this locus had limited effect against different isolates in other environments. The remaining 19 QTL represented single MTA effective against different isolates in specific environments (Figure 3 and Table 6). Interestingly, multiple loci on chromosomes 1B, 5A, 5B, and 6A were detected and dependent on the isolates used in a particular environment. For instance, 1B had four distinct QTL (8.56 Mbp: 205.68 Mbp to 307.43 Mbp; 586.30 Mbp; and 645.57 Mbp) detected in three environments, 5A had two QTL detected in two environments (588.37 Mbp and 609.87 Mbp), 6A had four QTL detected in three environments (11.11 Mbp; 16.56 Mbp; 17.66 to 17.91 Mbp; and 610.20 Mbp), and 5B had three QTL detected in four environments (514.93 Mbp; 539.46 Mbp; and 617.14 Mbp; Table 6). Phenotypic variance accounted by each SNP ranged from 7 to 10% and allele effects on reducing disease score ranged from 7.47 to 24.45% (Table 6). Therefore, multiple loci on the same chromosome control SNB resistance with varying effects of genes and alleles on reducing disease severity within and between environments and isolates. Individual SNP alleles representative of each locus consistently showed significantly (*P* < 0.05) reduced PLAD across all environments when stacking of more than one allele (Supplementary Figure S4). Marker alleles for QTL were different for some SNP in each of the four resistant wheat genotypes (Table 6) having different parentage (Supplementary Table S1) with consistent low PLAD at two locations across 3 years (Table 4). The resistant wheat genotypes probably inherited different genes and alleles for SNB response.

**FIGURE 4:**
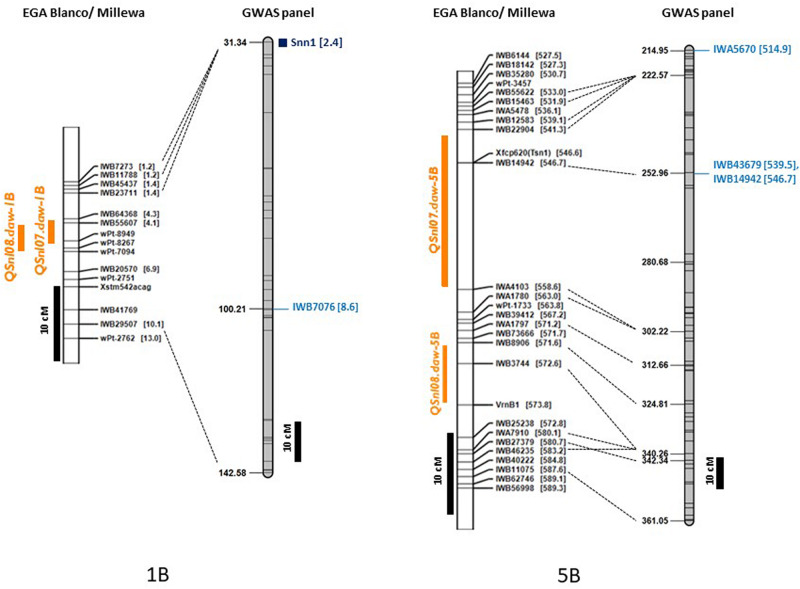
Comparison of maps for chromosomes 1B and 5B with genetic and physical position of QTL detected in the doubled haploid EGA Blanco/Millewa map (Francki et al., 2018) and GWAS panel. Physical location (in Mbp) of SNP are shown in square brackets with common markers anchoring EGA Blanco/Millewa and consensus map (Wang et al., 2014) shown by black dashed lines. QTL for SNB resistance detected in the DH map in 2017 and 2018 are shown in orange and SNP markers detecting QTL on the consensus map are shown in blue. Black bars represent centimorgan distances in the DH and consensus maps.

Single nucleotide polymorphic markers from the 90K Infinium SNP chip array used to genotype the GWAS panel enabled a direct comparison with QTL based on the physical map marker position (Alaux et al., 2018) and the wheat consensus map (Wang et al., 2014). Loci detected in this study, therefore, were compared to those previously reported for SNB response in other WA environments on chromosome 1B and 5B from a bi-parental doubled haploid (DH) mapping population derived from the parents EGA Blanco and Millewa (Francki et al., 2011; Francki et al., 2018). All of the 22 MTA identified for four QTL on chromosome 1B from GWAS analysis (Table 6) did not co-locate with the QTL, *QSnl07.daw-1B*, and *QSnl08.daw-1B* (4.3 Mbp to 6.9 Mbp) in the EGA Blanco/Millewa population (Figure 4). Therefore, it appears that regions on chromosome 1B contain multiple genes that respond to SNB in different environments and isolates. Similarly, the SNP marker *IWA5670* (at 514.9 Mbp) associated with SNB resistance in Katanning in 2016 was identified flanking the QTL *QSnl07.daw-5B* in the EGA Blanco/Millewa mapping population (Figure 4) and likely represents an alternative locus on chromosome 5B responding to SNB in specific environments and against diverse isolates. SNP markers *1WB43679* (at 539.5 Mbp) and *IWB14942* (at 546.7 Mbp) on chromosome 5B associated with SNB resistance at Northam and Manjimup in 2018, respectively, aligned to the QTL, *QSnl07.daw-5B* in the EGA Blanco/Millewa population (Figure 4) indicating similar regions harbor QTL detected in more than one environment.

### Comparison of MTA for SNB Resistance With Known SnTox-*Snn* Loci

**FIGURE 5:**
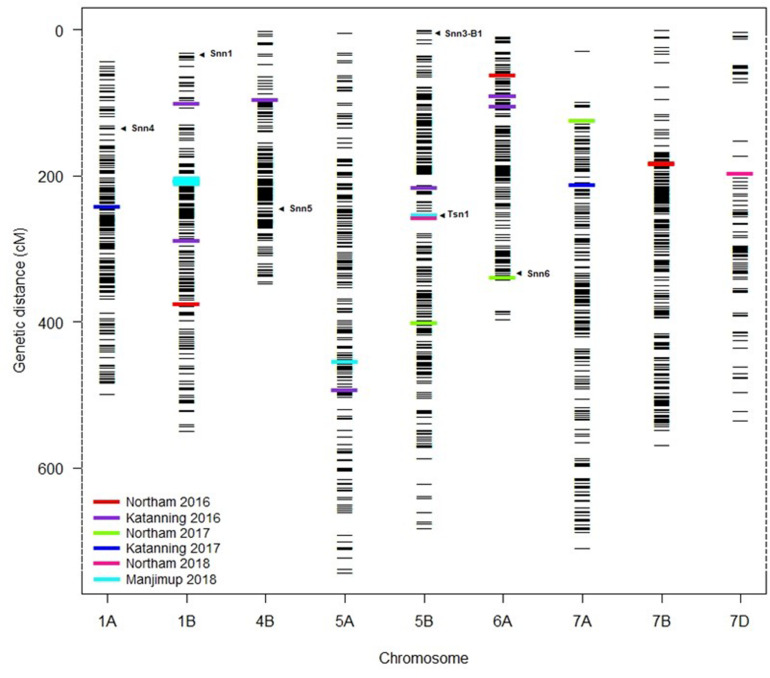
Comparison of QTL for SNB resistance with known *Snn* loci. Assignment of known *Tsn* and *Snn* loci and position of MTA detected in multiple environments in 2016–2018 on the genetic consensus map (Wang et al., 2014). Black horizontal lines represent the genetic locations (cM) of SNP markers used in GWAS analysis. Colored bars represent the MTA detected at two locations across 3 years (2016–2018). Arrows indicate the putative location of known *Snn* and *Tsn* loci.

The distribution of MTA across nine chromosomes were compared to locations for known *Snn* loci. SNP markers identified for *Tsn1* (Ruud et al., 2017; Ruud et al., 2019) *Snn1* and *Snn3-B1* (Ruud et al., 2017; Downie et al., 2018) loci were assigned directly to the genetic consensus map (Wang et al., 2014). The closest SSR markers linked to *Snn4* (Abeysekara et al., 2009), *Snn5* (Friesen et al., 2012), and *Snn6* (Gao et al., 2015) were cross-referenced with genetic maps consisting of SSR and SNP markers (Francki et al., 2018) to identify closely linked SNP as anchoring markers to assign *Snn4, Snn5*, and *Snn6* on the genetic consensus map (Wang et al., 2014). A total of six from eight known NE-host loci were mapped to nine chromosomes containing QTL detected across WA environments (Figure 5). The *Tsn1* locus represented by marker *fcp620* identified on 5B in the EGA Blanco/Millewa population was co-located with two SNP markers, *IWB14942* and *IWB43679*, at 252.96 cM on the consensus map (physical positions 546.70 Mbp and 539.46 Mbp, respectively) associated with SNB response in Northam and Manjimup 2018 (Figure 4). A QTL on 6A detected from the Northam 2018 environment only was within 6 cM of *Snn6* whereas the co-location of QTL on remaining chromosomes was not apparent with other known *Snn* loci (Figure 5).

**FIGURE 6:**
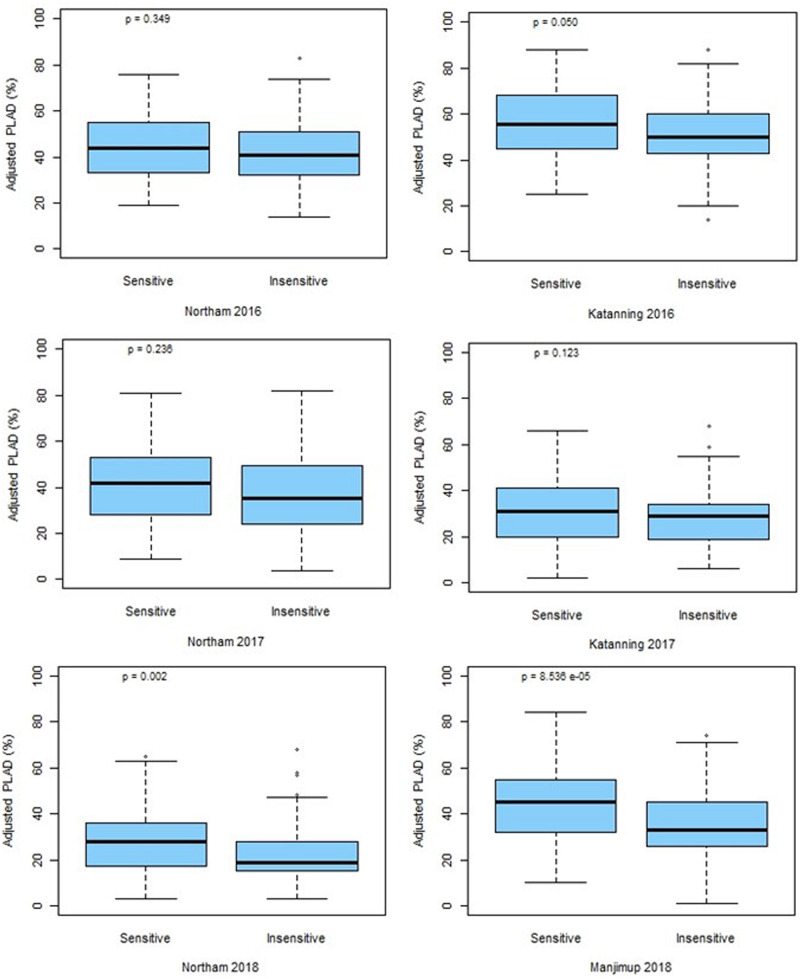
Allelic effects at *fcp620* locus at two locations across 3 years (2016–2018). *P*-values for significance are shown.

The marker for *Tsn1* (*fcp620*) was used to genotype the GWAS panel for sensitivity or insensitivity to SnToxA with 100% genotype identity with SNP marker *IWB14942* located at 546.70 Mbp (Figure 4). However, an additional SNP marker *IWB43679* located at 536.46 Mbp had only 69% genotype identity and, therefore, did not co-segregate with *Tsn1*. Box plot analysis confirmed the allelic effects of *fcp620* linked to *Tsn1* significantly (*P* < 0.05) reducing PLAD in only two environments, Northam 2018 and Manjimup 2018 (Figure 6) which corroborated similar allelic effects of *IWB14942* at these sites (Supplementary Figure S5).

## Discussion

The analysis of global wheat accessions for their response to APR against mixed isolates of SNB in WA field environments and cultivar-by-isolate-by-environment interactions are poorly understood. As environmental influences may either affect host gene response, virulence or aggressiveness of genetically diverse isolates, this study evaluated wheat accessions in two environments for three successive years against a mixture of isolates to expand our knowledge on SNB disease response and capture genotype-isolate-environment interactions. The outcomes of this study provided further insight to the complexity of these interactions and the underlying genetic control of SNB response for Australian wheat cultivars, inbred lines from CIMMYT, ICARDA, and some landraces.

*Parastagonospora nodorum* isolates have displayed a high degree of genetic diversity and the ability to evade plant host resistance (McDonald and Linde, 2002). Reports have shown that a wide range of *P. nodorum* isolates collected from different geographical regions of the United States can differ significantly in their aggressiveness and vary independently of the cultivar tested (Rufty et al., 1981; Scharen et al., 1985; Krupinsky, 1997), so it is plausible that isolates collected in diverse geographical regions of WA were genetically distinct with varying effects on disease development in regions other than the environment from where they were collected. Moreover, a number of environmental factors including climatic conditions for optimal spore germination, lesion development and sporulation or indeed the genetic basis influencing relative fitness and differential adaption of isolates to host cultivars can influence aggressiveness in different environments (Pariaud et al., 2009). SNB evaluation in this study detracted from using a single isolate as inoculum in field trials as it may neither be prominent in any season and environment, a strain assortment representative of a contemporary isolate, nor represent the breadth of diversity needed for identifying effective and robust SNB resistance. Instead, historical and contemporary SNB isolates presumed to be genetically distinct were used in mixed inoculum for evaluation and comparison of SNB response across wheat genotypes in different production environments. Highly significant genotype-by-environment interactions was consistent within and between years in this study and provided evidence that either alternative host genes, isolate virulence or aggressiveness, environment influences or a combination of these factors contribute to varying SNB response of wheat genotypes across environments. We anticipated consistent high correlation across genotypes in each year when inoculated with the same isolates under similar conditions in different trial locations but this was not evident particularly in 2016 and 2017 and across all years. Instead, both low to moderate correlation and high genotype-by-environment interactions were apparent. It is, therefore, reasonable to assume that different environments affect expression of alternative host genes against the same and different isolates. Conditions favoring isolate virulence or aggressiveness in one environment may also be different in another with a profound and variable effect on different host genes. Further studies to identify genetic factors controlling variability in isolate virulence and their interactions with alternative host genes would continue to unravel the biological and genetic complexity of genotype-by-environment-by-isolate effects on varying SNB disease response in wheat.

Despite varying SNB response in genotypes across environments, this study exploited the genetic diversity of breeding programs in Australia, Mexico, Middle East and landraces from discrete regions of the world to evaluate SNB response in WA environments. There were a total of 19 genotypes having robust resistance at two locations across 3 years against a total of 42 different isolates. Interestingly, EGA Blanco and 6HRWSN125, previously used as donor parents in QTL analysis (Shankar et al., 2008; Francki et al., 2011) showed consistent low PLAD scores indicating these lines have sustained expression of resistance particularly against contemporary isolates. Similarly, inbred lines from CIMMYT including 30ZJN09 and ZJN12 Qno 25, exhibited consistent stable low mean PLAD scores against historical and contemporary isolates in each year. Since EGA Blanco, 6HRWSN125, 30ZJN09, and ZJN12 Qno 25 have no common pedigree it appears, therefore, that SNB resistance could be derived from different genes or alleles with consistent SNB resistance in multiple environments. The different SNP alleles for QTL on chromosomes 1B, 4B, 5A, 5B, 7A, and 7D partly supports the inheritance of alternative genes or alleles while shared favorable alleles were found on 1B, 5B, and 6A. The four wheat genotypes with stable SNB resistance across multi-environments evaluated in this study are ideal donor parents for SNB resistance breeding.

A comparison of population structure using the full set of 20,563 SNP markers compared to the LD pruned datasets in this study showed no difference in population structure. The use of LD pruned datasets in genome-wide association studies were assumed to be favorable for avoiding deleterious effects caused by overrepresentation of markers for some regions of the genome or by artificially creating structure in the population caused by LD between markers rather than by true ancestry (supplementary note in Price et al., 2006). However, in practise, this is often not found to be the case and it is usually recommended to include a full set of cleaned marker data to accurately model more distant relationships and complex ancestry (Price et al., 2006; Eu-ahsunthornwattana et al., 2014). The PCA using the full set of 20,563 SNP markers confirmed that population structure was low and 15.6% of the total genetic variances was contributed by the first three principal components. Reduced population structure with a similar low total genetic variance was previously reported where wheat genotypes were predominantly sourced from one breeding program having extensive and reciprocal germplasm exchange with other programs resulting in a genetic bottleneck (Arruda et al., 2016). Similarly, CIMMYT wheat germplasm is widely distributed globally (Ortiz et al., 2008) so it is reasonable to conclude that reduced population structure of the panel in this study was a consequence of historical and frequent exchange of germplasm between breeding programs in Australia, CIMMYT and ICARDA. Given that population structure had low genetic variance, long-range LD blocks would be assumed for subgenomes of wheat. Although LD decay for the A and B subgenomes were similar to those reported for spring wheat populations (Chao et al., 2010; Bajgain et al., 2015), LD for the D subgenome was considerably higher in this study. The long-range LD decay to 50% of its original value at 26 cM in this study was similar to the D subgenome of hexaploid wheat reported to be 22 cM in a GWAS panel of germplasm sourced from CIMMYT and breeding programs in South America (Mora et al., 2015). Longer- range LD may be due to selection forces restricting recombination events required to retain favorable alleles in the D subgenome for broader adaption of CIMMYT, ICARDA, and Australian genotypes when breeding for geographically diverse environments. Longer-range LD in the D subgenome compared with the A and B subgenome of hexaploid wheat have also been attributed to recent introgressions and population bottlenecks (Chao et al., 2010).

Significant genotype-by-environment interactions within and between years supported conclusions that QTL may harbor concomitant disease-related genes that respond differently to isolate infection across environments (Francki et al., 2018). GWAS provided further evidence of multiple host genes on the same and different chromosomes that responded independently to different environments, isolates or both. Moreover, low to moderate correlations for PLAD scores across locations in successive years in this study were consistent to those previously reported for field-based SNB response (Shankar et al., 2008; Francki et al., 2011; Lu and Lillemo, 2014; Ruud et al., 2019) and QTL for SNB resistance detected in one WA environment may not necessarily have a significant effect on SNB response in another (Shankar et al., 2008; Francki et al., 2011). New loci on chromosomes 5A and 6A in this study provided further evidence of alternative genomic regions harbored disease-related genes that responded to specific WA environments and isolates.

Genome wide association studies for APR to SNB in multi-environments in Norway based on the iSelect Infinium 90K genotyping array (Ruud et al., 2019) enabled a tentative comparison of marker-trait associations across global field-based studies. SNP markers on chromosomes 1B (206.1 cM) and 5B (214.95 and 252.96 cM) detected in WA environments using the genetic consensus map location were associated with disease response either in Nordic field infection in 2011 or inoculation with individual Norwegian isolates NOR4 or 201618 (Ruud et al., 2019). Further comparative studies will determine whether these regions share common genes or whether distinctive disease-related gene clusters respond differently to pathogen infection across diverse environments as proposed by Francki et al. (2018). SNP markers for the remaining QTL detected in this study, however, did not correspond to other QTL in Nordic environments or isolates (Ruud et al., 2019; Lin et al., 2020) indicating that in some instances, alternative loci probably respond to SNB in different environments and against isolates from diverse global origins.

The co-location of QTL with the position of known SnTox-*Snn* and *Tsn* loci provides a means to identify the role of NE-host interactions in response to SNB disease in the field. The marker *fcp620* (co-segregated with *IWB14942*) on chromosome 5B indicated that *Tsn1* was associated with SNB resistance but only at Northam and Manjimup in 2018 and not the remaining environments. Therefore, it appears that the SnToxA-*Tsn1* interaction is inconsistent across environments in WA and corroborates a similar conclusion drawn from a previous QTL study using a bi-parental mapping population (Francki et al., 2011). Moreover, QTL did not co-locate with other known *Snn* loci indicating that historical and contemporary SNB isolates from WA may secrete alternative effector proteins or environmental influences may have an effect on known host-NE interactions. The inconsistent association with several known NE host susceptibility loci across environments, the preponderance of environment-specific QTL and minimal common loci across QTL from global field-based studies makes it reasonable to assume that alternative and distinct biological mechanisms are largely influenced either by the environment, isolates or their interactions. Biological mechanisms other than known SnTox-*Snn* interactions, including a myriad of biochemical and physiological process during infection, penetration and colonization (Bellincampi et al., 2014; Presti et al., 2015) may have a significant bearing on host resistance and susceptibility.

Additional studies are needed to identify other possible genes involved in SNB response but would require extensive field evaluation across wider locations and contemporary isolates to elucidate their role in specific environments. In doing so, it would provide a comprehensive analysis of the role of multiple genes on chromosomal blocks that respond to SNB infection and provide further insights on environmental factors and isolates affecting different biological pathways. In the meantime, the challenge for breeding SNB resistance is to transfer chromosomal blocks from donor resistant parents to increase the probability of an individual gene contributing suitable SNB resistance in any specific environment and against contemporary isolates. EGA Blanco, 6HRWSN125, 30ZJN09, and ZJN12 Qno 25 would be logical donor parents and the SNP markers identified on chromosomes on 1B, 5A, 5B, and 6A in this study will be important for marker-assisted selection and tracking chromosomal blocks harboring resistance genes.

## Conclusion

The wheat response to SNB infections against historical and contemporary isolates under multi-environment field conditions showed considerable genotype-by-environment-by isolate interactions within and between years. Although phenotypic correlation between wheat genotypes sourced from Australian cultivars, inbred lines from CIMMYT and ICARDA and some landraces was low, four genotypes expressed stable phenotypes with consistently low SNB symptoms at two locations across three years and identified as suitable donor parents for breeding SNB resistant wheat for WA production environments. GWAS identified 20 QTL with the majority detected in only one environment, confirming the complex genetic control for SNB response and the role of different environments and diverse isolates on expression of minor host genes. It appears that a majority of SnTox-*Snn* interactions are not evident with SnToxA-*Tsn1* being variable in different Western Australian environments and SNB response may involve other multiple complex biological mechanisms.

## Data Availability Statement

All datasets generated for this study are included in the article/Supplementary Material. Disease response for 232 lines at 6 locations and genotype data for SNP markers associated with SNB resistance are available in Supplementary Table S6.

## Author Contributions

MF designed the experiments, contributed to data acquisition, analysis and interpretation, and a major contributor to writing the manuscript. EW contributed to data acquisition, analysis and interpretation, and a major contributor to writing the manuscript. CM and WM contributed to trial designs, planting, maintenance, and data acquisition. All authors read and approved the final manuscript.

## Conflict of Interest

The authors declare that the research was conducted in the absence of any commercial or financial relationships that could be construed as a potential conflict of interest.

## Footnotes

1 http://www.caigeproject.org.au/

2 https://genstat.kb.vsni.co.uk
