# Supplementary material for: Multi-Location Evaluation of Global Wheat Lines Reveal Multiple QTL for Adult Plant Resistance to Septoria Nodorum Blotch (SNB) Detected in Specific Environments and in Response to Different Isolates
Source: Front Plant Sci. 2020 Jun 10;11:771. doi: 10.3389/fpls.2020.00771 (PMC7325896; doi:10.3389/fpls.2020.00771)

Figure S1 Top three principal components of wheat genotypes in the GWAS panel after correcting for linkage disequilibrium using SNP markers filtered with $r^2 \leq 0.2$ and $r^2 \leq 0.1$. Principal components 1 and 2 are shown on the left and principal components 1 and 3 on the right. Different colours represent Australian cultivars, inbred lines from CIMMYT and ICARDA and landraces from discrete regions of the world **(A)** Analysis using 2,941 SNP after filtering with $r^2 \leq 0.2$ **(B)** Analysis using 1,142 SNP after filtering with $r^2 \leq 0.1$.

A

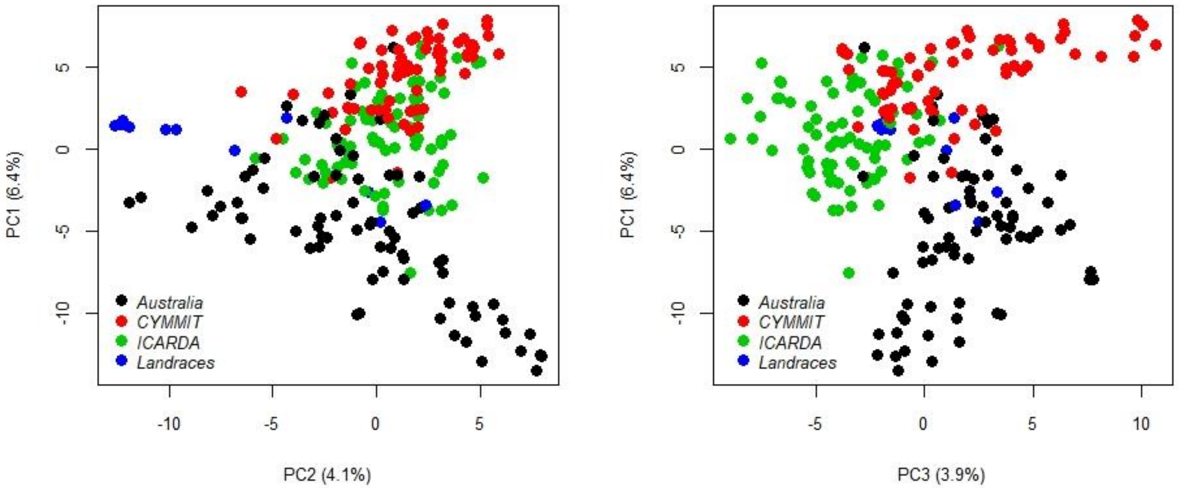

B

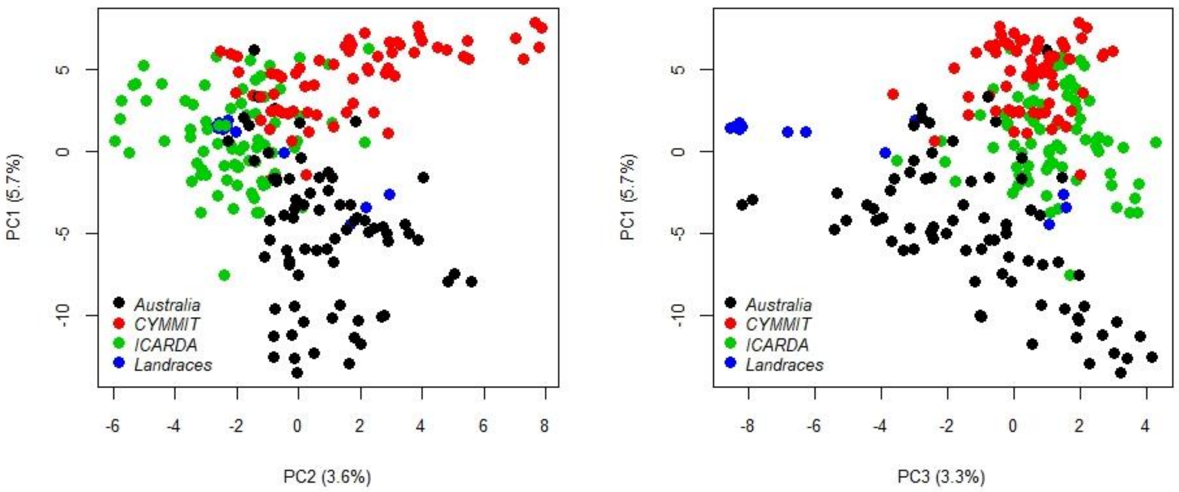

Supplement: Supplementary file 1 [file Data_Sheet_1.PDF]
