# Supplementary material for: Multi-Location Evaluation of Global Wheat Lines Reveal Multiple QTL for Adult Plant Resistance to Septoria Nodorum Blotch (SNB) Detected in Specific Environments and in Response to Different Isolates
Source: Front Plant Sci. 2020 Jun 10;11:771. doi: 10.3389/fpls.2020.00771 (PMC7325896; doi:10.3389/fpls.2020.00771)

Figure S3 Genome wide association scans for height scores for six individual environments in 2016-2018. Q-Q and Manhattan plots are shown to the left and right, respectively. Horizontal dotted lines in each environment represent suggestive (bottom), significant (middle) and highly significant (top) threshold levels for marker-trait associations.

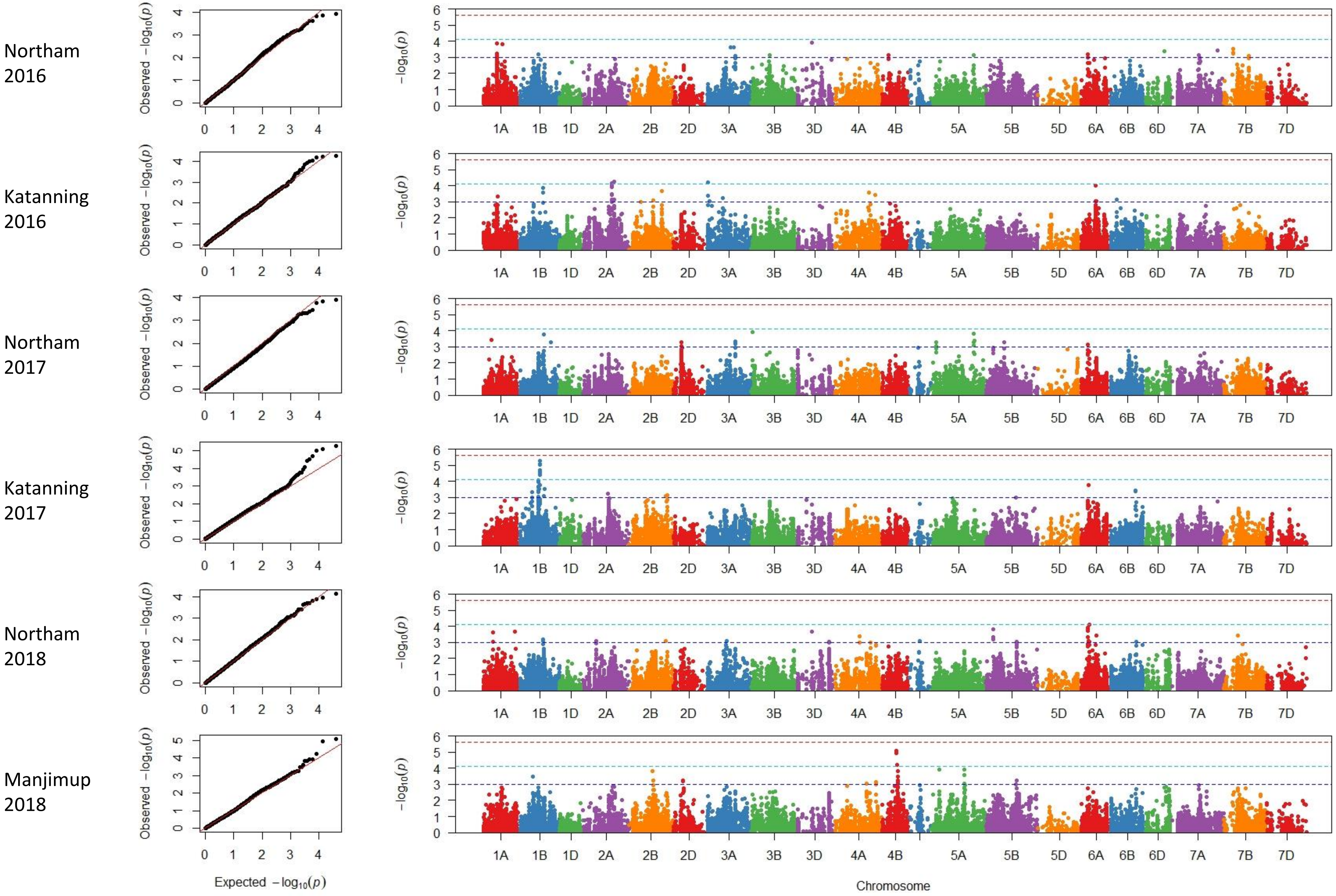

Supplement: Supplementary file 3 [file Data_Sheet_3.PDF]
