# Supplementary material for: Multi-Location Evaluation of Global Wheat Lines Reveal Multiple QTL for Adult Plant Resistance to Septoria Nodorum Blotch (SNB) Detected in Specific Environments and in Response to Different Isolates
Source: Front Plant Sci. 2020 Jun 10;11:771. doi: 10.3389/fpls.2020.00771 (PMC7325896; doi:10.3389/fpls.2020.00771)

Figure S4 Effects of increasing the number of alleles for reducing PLAD in each of the six environments (2016-2018). Markers used for determining the effects of allele stacking are described in Table 6.

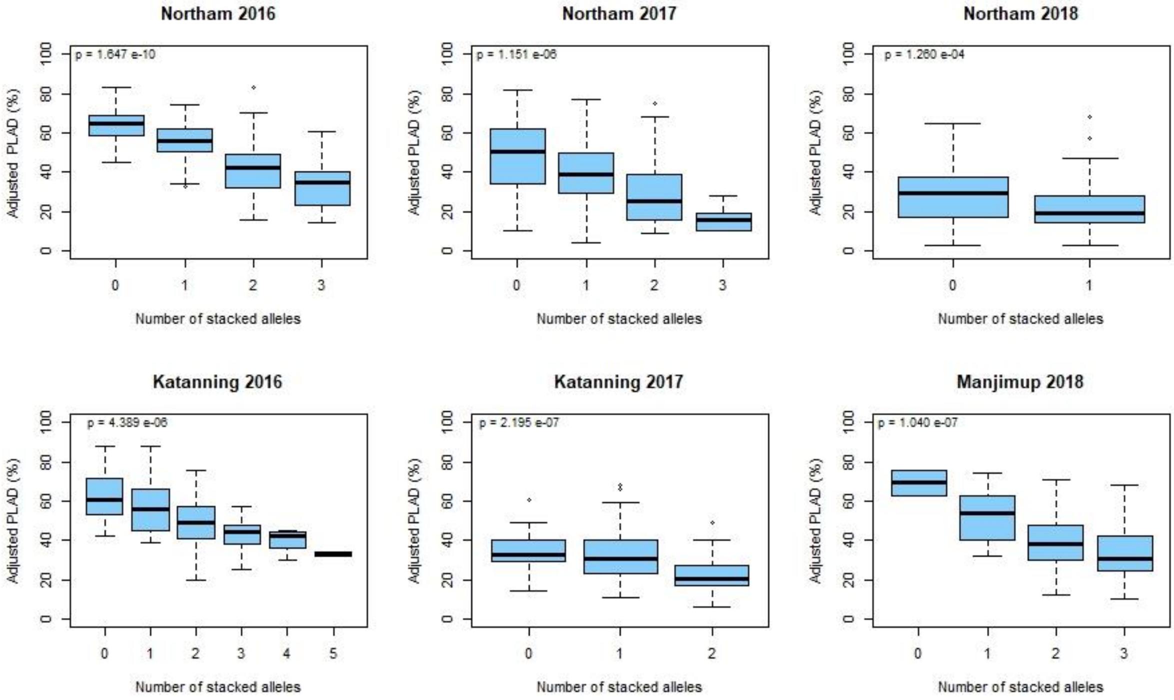

Supplement: Supplementary file 4 [file Data_Sheet_4.PDF]
