# Supplementary material for: Multi-Location Evaluation of Global Wheat Lines Reveal Multiple QTL for Adult Plant Resistance to Septoria Nodorum Blotch (SNB) Detected in Specific Environments and in Response to Different Isolates
Source: Front Plant Sci. 2020 Jun 10;11:771. doi: 10.3389/fpls.2020.00771 (PMC7325896; doi:10.3389/fpls.2020.00771)

**Figure S5** Effects of IWB14942 on reducing PLAD for each of the six environments (2016-2018). *P*-values for significance are shown.

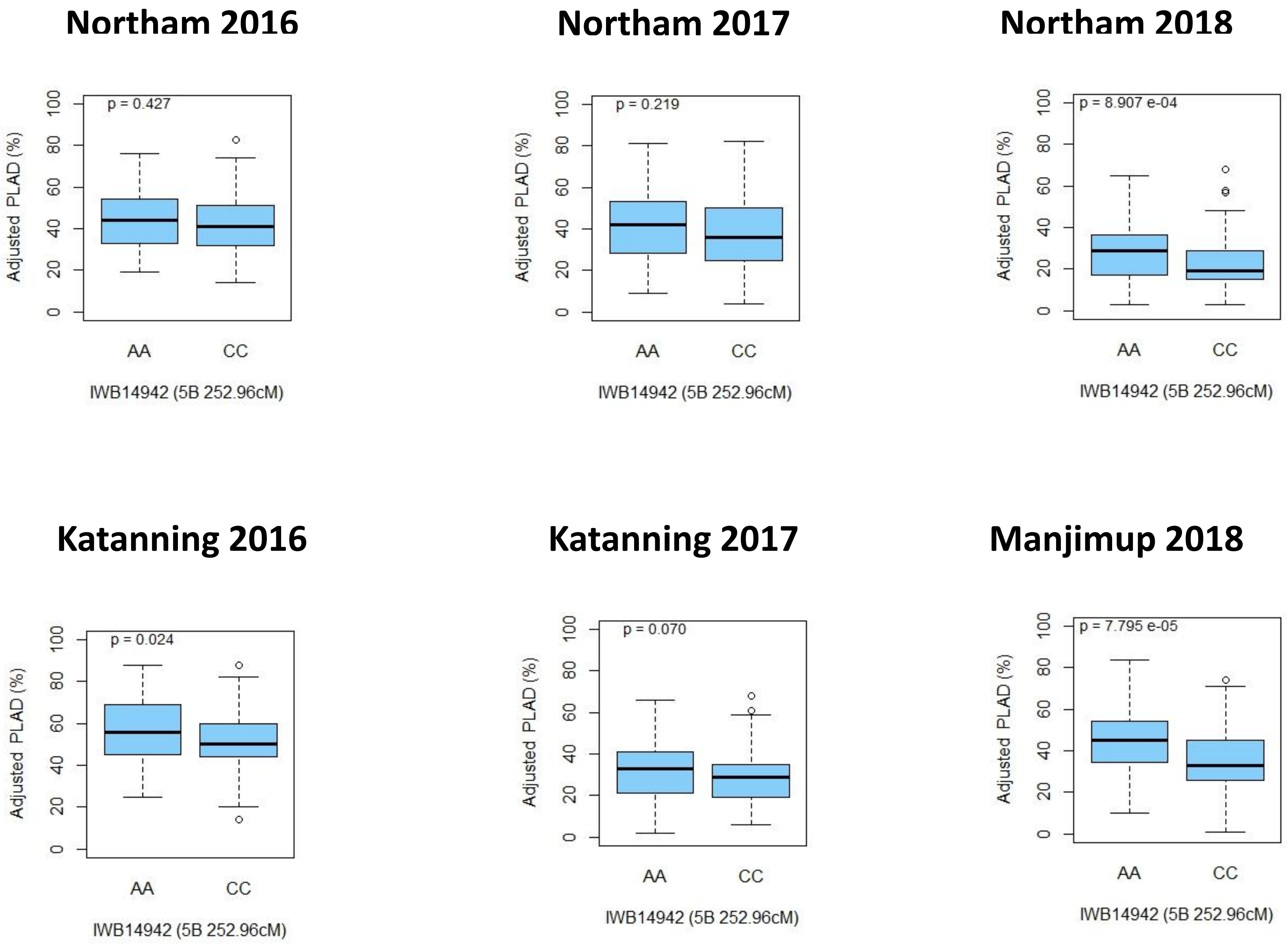

Supplement: Supplementary file 5 [file Data_Sheet_5.PDF]
