# Supplementary material for: Multi-Location Evaluation of Global Wheat Lines Reveal Multiple QTL for Adult Plant Resistance to Septoria Nodorum Blotch (SNB) Detected in Specific Environments and in Response to Different Isolates
Source: Front Plant Sci. 2020 Jun 10;11:771. doi: 10.3389/fpls.2020.00771 (PMC7325896; doi:10.3389/fpls.2020.00771)
Supplement: Supplementary file 6 [file Table_1.DOCX]

**Table S1** List of wheat genotypes from Australia, CIMMYT, ICARDA and others and their pedigree used in evaluation for SNB response in 2016-2018 and GWAS

| **LINE** | **PEDIGREE** |
| --- | --- |
|  |  |
| AUSTRALIAN |  |
|  |  |
| AJANA | BLADE/2*KULIN |
| AMERY | LR21-SRX/2*SHORTIM//3*BODALLIN |
| AROONA | LERMA ROJO-64//NORIN-10/BREVOR-14/3/3*ANDES(WW15)/4/RAVEN |
| ARRINO | 77W:660/ERADU |
| ATTILA | NORD-DESPREZ/VG-9144//KALYANSONA/BLUEBIRD/3/YACO/4/VEERY-5 |
| AUS20917 | N/A |
| AXE | (DH)RAC-875//EXCALIBUR/KUKRI/3/RAC-875//EXCALIBUR/KUKRI |
| BANKS | PWTH/(SIB)CONDOR//2*CONDOR |
| BARUNGA | BT-SCHOMBURGK/MOLINEUX |
| BAXTER | INIA-66/GAMUT//COOK/4/JUPATECO/3/LERMA-ROJO-64/SONORA-64-A//(SIB)TIMGALEN |
| BINNU | ARRINO/(Y89-4034) ERADU*4/VPM1 |
| BODALLIN | BOKAL/SIETE-CERROS-66 |
| BROOKTON | TORRES/CRANBROOK//76-W-596/CRANBROOK |
| BT SCHOMBURGK | HALBERD/AROONA//3*SCHOMBURGK |
| BUMPER | EXPRESS//PFAU/REEVES |
| CAMM | SPEAR*4//VPM-1/5*COOK |
| CARNAMAH | BOLSENA-1CH/77W:660 |
| CASCADES | AROONA*3//TADORNA/INIA66 |
| CHARA | BD-225/CD-87 |
| COBRA | (DER)WESTONIA |
| COCAMBA | AUS-10894/4*CONDOR |
| CORACK | WYALKATECHEM/SILVERSTAR |
| CRANBROOK | WREN,MEX//CIANO-67(SIB)/NOROESTE-66/3/ZAMBEZI |
| CRUSADER | (DH)SUNBROOK/H-45 |
| CUNDERDIN | CRANBROOK SISTER/SUNFIELD SISTER |
| DART | SUNBROOK/JANZ//KUKRI |
| DERRIMUT | VN-150/VN-715 |
| DRYSDALE | QUARRION/2*HARTOG |
| DUCULA | HUACAMAYO/TANORI-71-RESEL/3/ARTHUR*2/SIETE-CERROS-66//NACOZARI-76/4/ICTA-SARA-82 |
| EAGLE ROCK | SUNELG/2*BLADE |
| EGA BLANCO | BOBWHITE(SIB)/NARIS-HUNTSMAN//CRANBROOK/VICAM-71(83-Z-1288)/3/(IW-1266)PFAU |
| EGA BONNIE ROCK | SR9E.3*WARIGAL..3*AROONA (83Z:1048)/(82W:1097)3AG3.4*CONDOR..3*MILLEWA.3.BODALLIN |
| EGA BULLARING | 77-Z-893/81-Y-970 |
| EGA CASTLE ROCK | 3AG3.4*COOK/3*CASCADES |
| EGA2248 | 3AG3/3*HALBERD//4*TINCURRIN |
| ENVOY | N/A |
| ERADU | CIANO-67/GAMENYA |
| ESPADA | (DH)RAC-875/KRICHAUFF//EXCALIBUR/KUKRI/3/RAC-875/KRICHAUFF/4/RAC-875//EXCALIBUR/KUKRI |
| EXCALIBUR | RAC-177(SR26)/UNICULM-492//RAC-311-S |
| GLADIUS | (DH)RAC-875/KRICHAUFF//EXCALIBUR/KUKRI/3/RAC-875/KRICHAUFF/4/RAC-875//EXCALIBUR/KUKRI |
| HARTOG | VICAM-711/CIANO"S"/SIETECERROS/3/KLYNSN/BLUEBIRD |
| IMPALA | TEAL/C-93-8//9908 |
| KAUZ | JUPATECO-73/(SIB)BLUEJAY//URES-81 |
| KENNEDY | VEERY#5/HARTOG |
| KING ROCK | UNKNOWN |
| KUKRI | MADDEN/6*RAC-177//GRAJO/76-ECN-44 |
| KULIN | BODALLIN SIB//(HYDEN SIB)GAMENYA/INIA-66 |
| LANG | QT3765/SUNCO |
| MACE | WYALKATCHEM/STYLET//WYALKATCHEM |
| MILLEWA | SONORA-64/YAQUI-50-ENANO//GABOTO/II-8156 |
| MITRE | JANZ/BEULAH |
| SAPPHIRE | GBA-008/JANZ |
| SCEPTER | RAC1480/2*MACE |
| SCOUT | SUNSTATE/QH71-6//YITPI |
| SILVERSTAR | PAVON"S"/(TM56)COCAMBA SIB |
| SPITFIRE | DRYSDALE/KUKRI |
| STYLET | MOLINEUX/2*TRIDENT |
| SUNGUARD | SUN-289-E/SR2-JANZ |
| SUNTOP | TESTED AS SUN595B AGT INBRED/CIMMYT INBRED |
| TAMMARIN ROCK | SKOROSPELKA.4*LANCE:3*BODALLIN(81Y:970)/KALANNIE |
| TAMMIN | BODALLIN//ERADU SIB/XBVT223/3/ATLAS66/2*MADDEN |
| TROJAN | LPB 00LR000041/SENTINEL |
| VENTURA | SUNVALE/ROWAN |
| WALLUP | WYALKATCHEM/CHARA |
| WAWHT2046 | AUS22857/KULIN/BLADE |
| WAWHT2074 | UNKNOWN |
| WESTONIA | SPICA/TIMGALEN(QT2085-20)/TOSCA(CO1190-203)//(84W127-501)CRANBROOK:JACUP*2/BOBWHITE |
| WYALKATCHEM | MACHETE//(84W129-504)GUTHA/JACUP*2(11ISEPTON135)IASSUL/H567-71 |
| YANDANOOKA | CALINGIRI/WAWHT-1137//38-W-386443 |
| YARRALINKA | MENGAVI/SIETE-CERROS-66/3/MENGAVI/SIETE-CERROS-66//CRIM/4/COMBINATION-III/2*WARIGAL |
| YOUNG | VPM-1/3*BEULAH//SILVERSTAR |
|  |  |
| ICARDA |  |
|  |  |
| 03:ZIZ12 | TEVEE-3/SHUHA-20//SERI 82/SHUHA'S' |
| 04:ZIZ13 | TRACHA'S'//CMH76-252/PVN'S' |
| 05:ZIZ13 | CNDO/R143//ENTE/MEXI/3/AEGILOPSSQUARROSA(TAUS)/4/WEAVER/5/MYNA/VUL |
| 07:ZIZ13 | ZEMAMRA-5/SOMAMA-3 |
| 08:ZIZ13 | MUNIA//CHEN/ALTAR 84/3/CHEN/AEGILOPS SQUARROSA (TAUS)//BCN/4/MARCHOUCH-8 |
| 09:ZIZ13 | CHAM-6/SHUHA-14//QAFZAH-21 |
| 104:ZIZ13 | HIDDAB/FLORKWA-2 |
| 105:ZIZ13 | HAMAM-5/OUKAB-2//TAZA-1 |
| 106:ZIZ13 | HUBARA-3*2/SHUHA-4 |
| 109:ZIZ13 | QAFZAH-20/QIMMA-5//FERROUG-3 |
| 110:ZIZ13 | LAKTA-1/2*TAZA-2 |
| 111:ZIZ13 | ATENA-1/GAMDOW-3/3/MON'S'/ALD'S'//ALDAN'S'/IAS58 |
| 112:ZIZ13 | CHAM-6/PERW//MILAN/PASTOR/3/CHAM-6/PERW |
| 115:ZIZ13 | HUBARA-8/3/MUNIA/ALTAR 84//MILAN/4/ANGI-2 |
| 116:ZIZ12 | INQALAB 91/CMSW94WM00188S-0300M-0100Y-0100M-13Y-4M-0Y-0AP |
| 12:ZIZ13 | IZAZ-2/TEVEE-2/4/NAI60/HN7//SX/3/JUN'S' |
| 122:ZIZ13 | PASTOR-2*2//ACSAD 685/ACSAD 639 |
| 123:ZIZ13 | QAFZAH-23/SOMAMA-3//GOUMRIA-3 |
| 124:ZIZ12 | CMT/ALD//ZARZOUR/5/AU//KAL/BB/3/BON/4/HPO |
| 126:ZIZ13 | ATRIS-10/4/PASTOR/3/KAUZ*2/OPATA//KAUZ/5/CHAM-6/TUI'S' |
| 127:ZIZ13 | TAZA-2/LAKTA-5//HAAMA-11 |
| 13:ZIZ12 | PAVON 76/HAMAM-4/4/YACO/PBW65/3/KAUZ*2/TRAP//KAUZ |
| 13:ZIZ13 | IZAZ-11/ATTILA-7/4/OPATA/BOW//BAU/3/OPATA/BOW |
| 130:ZIZ13 | HUBARA-5/ANGI-1 |
| 131:ZIZ13 | HUBARA-5/PASTOR-2 |
| 136:ZIZ13 | ANGI-2/HUBARA-3 |
| 137:ZIZ13 | ATTILA-7/SOMAMA-3 |
| 141:ZIZ13 | BJY/COC//PRL/BOW/3/BLOYKA-1 |
| 142:ZIZ13 | KAUZ'S'/BOCRO-3//ANGI-2 |
| 145:ZIZ13 | CONDOR//SHUHA-6/CONDOR |
| 148:ZIZ13 | HUBARA-15/CATBIRD//PASTOR-2 |
| 149:ZIZ13 | HUBARA-5/3/SHA3/SERI//SHA4/LIRA/4/QAFZAH-21 |
| 151:ZIZ13 | HUBARA-1/3/MUNIA/CHTO//MILAN/4/GOUMRIA-8 |
| 152:ZIZ13 | HUBARA-5/PASTOR-2 |
| 154:ZIZ13 | KAUZ'S'/FLORKWA-1//GOUMRIA-3 |
| 156:ZIZ13 | FLORKWA-2/6/SAKER'S'/5/RBS/ANZA/3/KVZ/HYS//YMH/TOB/4/BOW'S'/7/DAJAJ-6 |
| 157:ZIZ13 | HUBARA-3/SHUHA-4//PASTOR-2 |
| 158:ZIZ13 | KATILA-17/DEEK-2/8/VEE'S'/7/CEBECO148/3/RON/CHA//BB/NOR67/5/HK/38MA/4/4777//REI/Y/3/KT/6/TUCAN'S' |
| 159:ZIZ13 | HUBARA-2/QAFZAH-21//DOVIN-2 |
| 16:ZIZ13 | ZEMAMRA-8/3/SHA3/SERI//SHA4/LIRA/4/QAFZAH-21 |
| 20:ZIZ12 | SERI 82/SHUHA'S'//CM84655-02AP-300AP-300L-3AP-300L-3AP-0L-0AP |
| 21:ZIZ13 | ZEMAMRA-5/ZEMAMRA-5 |
| 22:ZIZ12 | BANA-1///NS732/HER//SHUHA-15 |
| 23:ZIZ13 | TINAMOU-2//TEVEE-1/SHUHA-6 |
| 31:ZIZ13 | KARAWAN-1/TALLO 3//JADIDA-2 |
| 32:ZIZ12 | FERROUG-2/POTAM*2KS811261-8//ZEMAMRA-8 |
| 33:ZIZ12 | MON'S'/ALD'S'//ALDAN'S'/IAS58/3/SAFI-1/4/ZEMAMRA-1 |
| 33:ZIZ13 | SOUS-1/ARREHANE |
| 36:ZIZ13 | TEVEE-1/SHUHA-6//MASSIRA |
| 37:ZIZ12 | DORADE/KAPSW |
| 38:ZIZ13 | SHUHA-2//NS732/HER/3/AGUILAL |
| 41:ZIZ13 | MASSIRA/SAFI-1 |
| 43:ZIZ13 | TEVEE-1/STAR'S'/3/ACHTAR*3//KANZ/KS85-8-4 |
| 45:ZIZ12 | HUBARA-8///MON'S'/ALD'S'//BOW'S' |
| 45:ZIZ13 | SHUHA-7/SHUHA-14//MARHOUCH*4/SAADA |
| 47:ZIZ13 | ACHTAR//ATTILA-1/NS732/HER |
| 48:ZIZ12 | QAFZAH-33/ICW84.0074-09AP-300L-1AP-300L-8AP-0L-0AP |
| 48:ZIZ13 | MOUKA-4*2/4/KEA'S'/3/MN72252//HD2169/BOW'S' |
| 50:ZIZ13 | QAFZAH-21/OUEDZEM-1 |
| 51:ZIZ12 | SERI 82/SHUHA'S'//CM85295-0101TOPY-2M-0Y-0M-3Y-0M-0AP |
| 52:ZIZ12 | ICW91.0145-4AP-0TS-2AP-0L-0AP/4/NAI60/HN7//SX/3/JUN'S' |
| 53:ZIZ12 | ICW91.0145-4AP-0TS-2AP-0L-0AP/QAFZAH-33 |
| 53:ZIZ13 | MEXIPAK/FLORKWA-2 |
| 54:ZIZ13 | JUP/BJY//URES |
| 55:ZIZ13 | CHAM-4/SHUHA'S'/6/2*SAKER/5/RBS/ANZA/3/KVZ/HYS//YMH/TOB/4/BOW'S' |
| 56:ZIZ13 | SHUHA-7//SERI 82/SHUHA'S' |
| 57:ZIZ13 | ND/VG9144//KAL/BB/3/YACO/4/VEE#5 |
| 61:ZIZ12 | NS732/HER//SAADA///SAADA |
| 61:ZIZ13 | BOCRO-2/ABTIN-1 |
| 66:ZIZ13 | ZEMAMRA-1/2*SOMAMA-3 |
| 69:ZIZ13 | SHUHA-4/FLORKWA-4//HUBARA-3 |
| 72:ZIZ12 | ACHTAR/IG 132176 |
| 75:ZIZ13 | HUBARA-3*2/SHUHA-4 |
| 80:ZIZ12 | BT1735/ACHTAR//ASFOOR-1 |
| 81:ZIZ13 | HUBARA-3*2/SHUHA-4 |
| 88:ZIZ13 | ATTILA-7/KATILA-12 |
| 90:ZIZ13 | HD2281/PVN/3/KAUZ*2/TRAP//KAUZ/4/AALAAL-1 |
| 91:ZIZ13 | KAUZ//TRAP#1/BOW/3/QAFZAH-21 |
| 96:ZIZ13 | FLORKWA-2/6/SAKER'S'/5/RBS/ANZA/3/KVZ/HYS//YMH/TOB/4/BOW'S'/7/DAJAJ-6 |
| 98:ZIZ13 | HIDDAB/ATTILA-7 |
|  |  |
| CIMMYT |  |
|  |  |
| 30ZJN09 | CS/TH.CU//GLEN/3/ALD/PVN/4/NINGMAI NO.4/OLESON//ALD/YANGMAI NO.4 |
| 6HRWSN098 | N/A |
| 6HRWSN125 | GOLDEN-VALLEY/AZTECA-67//MUSALA/3/DODO/4/BOBWHITE |
| Babax | BOBWHITE/NACOZARI-76//VEERY/3/BLUEJAY/COCORAQUE-75 |
| Berkut | IRENA/BAVIACORA-M-92//PASTOR |
| Inia66 | LERMA-ROJO-64/SONORA-64 |
| Pfau | HORK(SIB)/YAMHILL//KALYANSONA/BLUEBIRD |
| Sokoll | PASTOR/3/ALTAR-84/AE.SQ(TR.TA)//OPATA-M-85 |
| ZEE10Qno133 | SUN371A*2/3/CHEN/AE.SQ//WEAVER |
| ZEE10Qno77 | VEE/MJI//2*TUI/3/2*PASTOR/4/BERKUT/5/PFAU/MILAN |
| ZEE10Qno95 | FRET2//SKAUZ*2/FCT/3/FILIN/2*PASTOR |
| ZEE10Qno96 | ATTILA*2/9/KT/BAGE//FN/U/3/BZA/4/TRM/5/ALDAN/6/SERI/7/VEE#10/8/OPATA/10/CHEN/AEGILOPS SQUARROSA (TAUS)//FCT/3/2*WEAVER/11/PRL/2*PASTOR |
| ZIZ11Qno81 | PEWIT3/4/ 67109/FRD//P101/FW71002/3/2*VEE#6 |
| ZJN10Qno12 | N/A |
| ZJN10Qno3 | N/A |
| ZJN10Qno8 | N/A |
| ZJN10Qno9 | N/A |
| ZJN11Qno104 | CROC_1/AE.SQUARROSA (213)//PGO/3/CMH81.38/2*KAUZ/4/BERKUT |
| ZJN11Qno105 | OR 9437534/SOKOLL//SOKOLL |
| ZJN11Qno106 | MTRWA92.161/PRINIA/5/SERI*3//RL6010/4*YR/3/PASTOR/4/BAV92 |
| ZJN11Qno21 | KS82142/2*WBLL1 |
| ZJN11Qno22 | KS82142/2*WBLL1 |
| ZJN11Qno23 | KS82142/2*WBLL1 |
| ZJN11Qno24 | KS82142/2*WBLL1 |
| ZJN11Qno25 | KS82W418/SPN/3/CHEN/AE.SQ//2*OPATA/4/FRET2 |
| ZJN11Qno29 | PASTOR//HXL7573/2*BAU/3/SOKOLL/WBLL1 |
| ZJN11Qno46 | SOKOLL//SLVS/PASTOR/3/ATTILA*2//CHIL/BUC |
| ZJN11Qno47 | SOKOLL//W15.92/WBLL1 |
| ZJN11Qno7 | VEE/MJI//2*TUI/3/2*PASTOR/4/BERKUT |
| ZJN11Qno70 | VEE/MJI//2*TUI/3/2*PASTOR/4/BERKUT |
| ZJN12Qno18 | CNO79//PF70354/MUS/3/PASTOR/4/BAV92/5/FRET2/KUKUNA//FRET2/6/MILAN/KAUZ//PRINIA/3/BAV92 |
| ZJN12Qno25 | CNO79//PF70354/MUS/3/PASTOR/4/BAV92/5/ATTILA*2/PBW65/6/PBW343*2/TUKURU |
| ZJN12Qno9 | HUANIL//2*WBLL1*2/KUKUNA |
| ZVS07Qno227 | MILAN/SHA7/3/CROC_1/AE.SQUARROSA (224)//OPATA |
| ZVS09Qno123 | DVERD_2/AE.SQUARROSA (214)//2*BCN/3/CALINGIRI |
| ZVS09Qno124 | DVERD_2/AE.SQUARROSA (214)//2*BCN/3/CALINGIRI |
| ZVS09Qno125 | DVERD_2/AE.SQUARROSA (214)//2*BCN/3/CALINGIRI |
| ZVS09Qno133 | MILAN/DUCULA/4/CROC_1/AE.SQUARROSA (205)//KAUZ/3/SASIA |
| ZVS09Qno191 | CALINGIRI/SOKOLL |
| ZVS09Qno33 | SHARPSHOOTER/BRBT2 |
| ZVS09Qno92 | N/A |
| ZWB11Qno124 | TRCH/HUIRIVIS #1 |
| ZWB11Qno172 | WAXWING/4/BL 1496/MILAN/3/CROC_1/AE.SQUARROSA (205)//KAUZ/5/FRNCLN |
| ZWB11Qno56 | N/A |
| ZWB11Qno95 | MUNAL #1/FRANCOLIN #1 |
| ZWC08Qno300 | BETTY/3/CHEN/AE.SQ//2*OPATA |
| ZWE08Qno109 | ATTILA*2/PBW65//BERKUT |
| ZWE10Qno30 | SOKOLL//SUNCO/2*PASTOR |
| ZWW09Qno125 | MTRWA92.161/PRINIA/5/SERI*3//RL6010/4*YR/3/PASTOR/4/BAV92 |
| ZWW09Qno157 | BERKUT/EXCALIBUR |
| ZWW09Qno176 | SOKOLL/EXCALIBUR |
| ZWW09Qno177 | SOKOLL/EXCALIBUR |
| ZWW09Qno40 | CROC_1/AE.SQUARROSA (213)//PGO/3/CMH81.38/2*KAUZ/4/BERKUT |
| ZWW09Qno43 | CHEN/AEGILOPS SQUARROSA (TAUS)//BCN/3/BAV92/4/BERKUT |
| ZWW09Qno59 | KLDR/PEWIT1//MILAN/DUCULA |
| ZWW09Qno72 | SOKOLL/EXCALIBUR |
| ZWW10Qno104 | SOKOLL//FRTL/2*PIFED |
| ZWW10Qno127 | CUNNINGHAM/4/SNI/TRAP#1/3/KAUZ*2/TRAP//KAUZ |
| ZWW10Qno128 | ESDA/KKTS |
| ZWW10Qno133 | N/A |
| ZWW10Qno139 | SOKOLL*2/4/CHEN/AEGILOPS SQUARROSA (TAUS)//FCT/3/STAR |
| ZWW10Qno140 | SOKOLL*2/4/CHEN/AEGILOPS SQUARROSA (TAUS)//FCT/3/STAR |
| ZWW10Qno155 | PASTOR*2/BAV92/5/FRET2*2/4/SNI/TRAP#1/3/KAUZ*2/TRAP//KAUZ |
| ZWW10Qno157 | TUKURU/4/CROC_1/AE.SQUARROSA (224)//YACO/3/MUNIA/5/BABAX/LR42//BABAX |
| ZWW10Qno29 | NSM*4/14-2//FRTL/2*PIFED/3/VORB |
| ZWW10Qno31 | BABAX/LR42//BABAX/3/BABAX/LR42//BABAX/4/T.DICOCCON PI94625/AE.SQUARROSA (372)//3*PASTOR/5/T.DICOCCON PI94625/AE.SQUARROSA (372)//3*PASTOR |
| ZWW10Qno5 | POTCH 93/4/MILAN/KAUZ//PRINIA/3/BAV92/5/MILAN/KAUZ//PRINIA/3/BAV92 |
| ZWW10Qno51 | ACHTAR/4/MILAN/KAUZ//PRINIA/3/BAV92 |
| ZWW10Qno52 | FDC36//ATTILA*2/PBW65 |
| ZWW10Qno60 | MILAN/KAUZ//PRINIA/3/BAV92/4/ATTILA/BAV92//PASTOR/5/CNO79//PF70354/MUS/3/PASTOR/4/BAV92 |
| ZWW10Qno76 | SOKOLL*2/ROLF07 |
| ZWW10Qno79  OTHER | GK ARON/AG SECO 7846//2180/4/2*MILAN/KAUZ//PRINIA/3/BAV92 |
| 040HAT10 | PAKISTAN LANDRACE |
| 055HAT10 | PAKISTAN LANDRACE |
| 057HAT10 | PAKSITAN LANDRACE |
| 058HAT10 | PAKISTAN LANDRACE |
| 070HAT10 | PAKISTAN LANDRACE |
| 082HAT10 | PAKISTAN LANDRACE |
| 122HAT10 | PAKSITAN LANDRACE |
| 125HAT10 | PAKSITAN LANDRACE |
| 135HAT10 | JORDAN LANDRACE |
| 170HAT10 | TAJIKISTAN LANDRACE |
| JingHing No5 | N/A |
