# Supplementary material for: Multi-Location Evaluation of Global Wheat Lines Reveal Multiple QTL for Adult Plant Resistance to Septoria Nodorum Blotch (SNB) Detected in Specific Environments and in Response to Different Isolates
Source: Front Plant Sci. 2020 Jun 10;11:771. doi: 10.3389/fpls.2020.00771 (PMC7325896; doi:10.3389/fpls.2020.00771)
Supplement: Supplementary file 7 [file Table_2.DOCX]

| **Isolates for 2016 SNB trials** | | | **Isolates for 2017 SNB trials** | | | **Isolates for 2018 SNB trials** | | |
| --- | --- | --- | --- | --- | --- | --- | --- | --- |
| Isolate | Year | Location | Isolate | Year | Location | Isolate | Year | Location |
| WAC 13205 | 2006 | Jarrahdale | WAC8635 | 1994 | Unknown | **WAC13077** | 2005 | Geraldton |
| **WAC 13206** | 2008 | South Perth | WAC13077 | 2005 | Geraldton | **WAC13206** | 2008 | South Perth |
| WAC 13404 | 2010 | South Perth | **WAC13206** | 2008 | South Perth | **WAC13524** | 2011 | South Perth |
| WAC 13526 | 2011 | South Perth | WAC13405 | 2010 | South Perth | **WAC 13690** | 2014 | Dangara |
| WAC 13530 | 2011 | South Perth | WAC13528 | 2011 | South Perth | **WAC 13741** | 2014 | Katanning |
| WAC 13616 | 2012 | Geraldton | **WAC13524** | 2011 | South Perth | **WAC13872** | 2015 | Geraldton |
| WAC 13666 | 2013 | Dongara | **WAC13667** | 2013 | Dongara | **WAC13957** | 2016 | South Perth |
| **WAC 13667** | 2013 | Dongara | **WAC13741** | 2014 | Katanning | **WAC13967** | 2016 | Merredin |
| WAC 13691 | 2014 | Dongara | **WAC13690** | 2014 | Dongara | **WAC13969** | 2016 | Wongan Hills |
| WAC 13740 | 2014 | Badgingarra | **WAC13872** | 2015 | Geraldton | WAC13979 | 2017 | Wongan Hills |
| WAC 13865 | 2015 | Kalannie | **WAC13873** | 2015 | Nungarin | WAC14056 | 2017 | Wongan Hills |
| WAC 13869 | 2015 | Wongan Hills | WAC13955 | 2016 | Northam | WAC14057 | 2017 | Wongan Hills |
| WAC 13870 | 2015 | Geraldton | **WAC13957** | 2016 | South Perth | WAC14058 | 2017 | Geraldton |
| WAC 13871 | 2015 | Geraldton | WAC13958 | 2016 | South Perth | WAC14059 | 2017 | Geraldton |
| **WAC 13872** | 2015 | Geraldton | WAC13959 | 2016 | South Perth | WAC14060 | 2017 | Geraldton |
| **WAC 13873** | 2015 | Nungarin | WAC13966 | 2016 | Merredin | WAC14061 | 2017 | Geraldton |
|  |  |  | **WAC13967** | 2016 | Merredin | WAC14062 | 2017 | Wongan Hills |
|  |  |  | WAC13968 | 2016 | Wongan Hills | WAC14066 | 2017 | Northam |
|  |  |  | **WAC13969** | 2016 | Wongan Hills | WAC14067 | 2017 | Northam |
|  |  |  | WAC13970 | 2016 | Wongan Hills |  |  |  |

**Table S2** *P. nodorum* isolate description used for field evaluation of wheat genotypes in 2016-2018. Year and geographical location of isolates collected in Western Australia is provided. Individual isolates used in successive years are highlighted in bold.
