# Supplementary material for: Multi-Location Evaluation of Global Wheat Lines Reveal Multiple QTL for Adult Plant Resistance to Septoria Nodorum Blotch (SNB) Detected in Specific Environments and in Response to Different Isolates
Source: Front Plant Sci. 2020 Jun 10;11:771. doi: 10.3389/fpls.2020.00771 (PMC7325896; doi:10.3389/fpls.2020.00771)
Supplement: Supplementary file 8 [file Table_3.DOCX]

**Table S3** Summary of SNP marker associated with heading date from two locations in three successive years (2016-2018). Shaded blocks represent SNP markers that are in strong LD when the bottom of the 90% D-prime confidence interval is greater than 0.70, and the top of the confidence interval is at least 0.98 as defined in Gabriel et al. (2002).

| **Environment** | **Chromosome** | **Marker** | **SNP^a^** | **Consensus map postion-cM** | **IGWSC-bp**^a^ | **R^2^** | **MAF^b^** | **Allele effect estimate %^c^** | ***p*-value** | **-log_10_(*p*)** |
| --- | --- | --- | --- | --- | --- | --- | --- | --- | --- | --- |
| Northam 2016 | 5B | IWB630 | A/**G** | 433.05 | 656,260,721 | 0.09 | 0.24 | 5.07 | 1.87E-05 | 4.73 |
|  | 5B | IWB6568 | **A**/G | 433.05 | 656,260,205 | 0.08 | 0.22 | -4.60 | 4.74E-05 | 4.32 |
|  | 5B | IWB9238 | A/**G** | 433.05 | 656,713,538 | 0.08 | 0.27 | 4.76 | 3.53E-05 | 4.45 |
|  | 5B | IWB25322 | A/**G** | 433.05 | 656,260,745 | 0.09 | 0.26 | 5.05 | 1.43E-05 | 4.85 |
|  | 5B | IWB26068 | **T**/C | 433.05 | 656,255,154 | 0.09 | 0.25 | -4.99 | 1.44E-05 | 4.84 |
|  | 5B | IWB35088 | T/**C** | 433.05 | 656,259,302 | 0.08 | 0.26 | 4.68 | 4.75E-05 | 4.32 |
|  | 5D | IWB63558 | **A**/G | 354.06 | 462,988,671 | 0.08 | 0.49 | -5.12 | 3.50E-05 | 4.46 |
| Katanning 2016 | 1B | IWB7144 | **T**/C | 355.42 | 637,826,181 | 0.10 | 0.48 | -5.64 | 6.06E-05 | 4.22 |
|  | 5B | IWB40926 | T/**C** | 323.81 | 571,203,472 | 0.10 | 0.28 | 7.24 | 7.33E-05 | 4.13 |
| Northam 2017 | 2D | IWA989 | A/**G** | 51.11 | 32,792,768 | 0.08 | 0.48 | 8.22 | 6.34E-05 | 4.20 |
| Katanning 2017 | NA |  |  |  |  |  |  |  |  |  |
| Northam 2018 | NA |  |  |  |  |  |  |  |  |  |
| Manjimup 2018 | 1B | IWB72443 | T/**C** | 428.89 | 664,302,556 | 0.10 | 0.05 | 12.35 | 6.47E-06 | 5.19 |

^a^IWGSC: IWGSC RefSeq v1.0, bp: base pairs.

^b^MAF: minor allele frequency.

^c^The effect estimates the difference between the average phenotypic values of the homozygous A genotype relative to the homozygous B genotype.

NA: No associations detected at -log_10_(*p*) > 4.12.
