# Supplementary material for: Multi-Location Evaluation of Global Wheat Lines Reveal Multiple QTL for Adult Plant Resistance to Septoria Nodorum Blotch (SNB) Detected in Specific Environments and in Response to Different Isolates
Source: Front Plant Sci. 2020 Jun 10;11:771. doi: 10.3389/fpls.2020.00771 (PMC7325896; doi:10.3389/fpls.2020.00771)
Supplement: Supplementary file 9 [file Table_4.DOCX]

**Table S4** Summary of SNP marker associated with plant height from two locations in three successive years (2016-2018). Shaded blocks represent SNP markers that are in strong LD when the bottom of the 90% D-prime confidence interval is greater than 0.70, and the top of the confidence interval is at least 0.98 as defined in Gabriel et al. (2002).

| **Environment** | **Chromosome** | **Marker** | **SNP^a^** | **Consensus map postion-cM** | **IGWSC-bp**^a^ | **R^2^** | **MAF^b^** | **Allele effect estimate %^c^** | ***p*-value** | **-log_10_(*p*)** |
| --- | --- | --- | --- | --- | --- | --- | --- | --- | --- | --- |
| Northam 2016 | NA |  |  |  |  |  |  |  |  |  |
| Katanning 2016 | 2A | IWB47526 | **A**/G | 385.17 | 691,228,261 | 0.09 | 0.23 | -4.73 | 6.75E-05 | 4.17 |
|  | 2A | IWB40126 | **A**/G | 413.64 | 691,217,702 | 0.10 | 0.24 | -4.83 | 5.46E-05 | 4.26 |
|  | 3A | IWB13256 | T/**G** | 47.94 | 8,686,977 | 0.09 | 0.13 | 6.07 | 6.11E-05 | 4.21 |
| Northam 2017 | NA |  |  |  |  |  |  |  |  |  |
| Katanning 2017 | 1B | IWB9918 | **A**/G | 287.28 | 587,048,100 | 0.10 | 0.47 | -4.60 | 5.15E-06 | 5.29 |
|  | 1B | IWB56553 | T/**C** | 287.98 | 587,984,815 | 0.10 | 0.45 | 4.31 | 1.94E-05 | 4.71 |
|  | 1B | IWB27934 | A/**G** | 287.98 | 587,985,842 | 0.10 | 0.37 | 4.91 | 2.91E-05 | 4.54 |
| Northam 2018 | NA |  |  |  |  |  |  |  |  |  |
| Manjimup 2018 | 4B | IWB6961 | **T**/C | 193.52 | 475,806,782 | 0.09 | 0.05 | -10.75 | 1.18E-05 | 4.93 |
|  | 4B | IWB72211 | **A**/G | 193.52 | 475,806,882 | 0.09 | 0.06 | -10.74 | 8.29E-06 | 5.08 |
|  | 4B | IWB26339 | **T**/C | 195.17 | 479,154,233 | 0.08 | 0.07 | -9.30 | 6.24E-05 | 4.21 |

^a^IWGSC: IWGSC RefSeq v1.0, bp: base pairs.

^b^MAF: minor allele frequency.

^c^The effect estimates the difference between the average phenotypic values of the homozygous A genotype relative to the homozygous B genotype.

NA: No associations detected at -log_10_(*p*) > 4.12.
