# Supplementary material for: Multi-Location Evaluation of Global Wheat Lines Reveal Multiple QTL for Adult Plant Resistance to Septoria Nodorum Blotch (SNB) Detected in Specific Environments and in Response to Different Isolates
Source: Front Plant Sci. 2020 Jun 10;11:771. doi: 10.3389/fpls.2020.00771 (PMC7325896; doi:10.3389/fpls.2020.00771)
Supplement: Supplementary file 10 [file Table_5.DOCX]

**Table S5** Genome and chromosome comparison between the full data set containing 20,563 SNP and a subset containing 1,142 SNP (LD pruned r^2^≤0.1) . **(A)** Total number of SNP for each chromosome **(B)** Distances between SNP.

**A**

| **Chromosome** | **Full SNP set** | **LD pruned r^2^≤0.1** |  |  |  |  |  |  |
| --- | --- | --- | --- | --- | --- | --- | --- | --- |
| 1A | 1214 | 50 |  |  |  |  |  |  |
| 1B | 1907 | 70 |  |  |  |  |  |  |
| 1D | 492 | 29 |  |  |  |  |  |  |
| 2A | 1151 | 55 |  |  |  |  |  |  |
| 2B | 1844 | 94 |  |  |  |  |  |  |
| 2D | 687 | 34 |  |  |  |  |  |  |
| 3A | 990 | 61 |  |  |  |  |  |  |
| 3B | 1432 | 61 |  |  |  |  |  |  |
| 3D | 298 | 29 |  |  |  |  |  |  |
| 4A | 982 | 54 |  |  |  |  |  |  |
| 4B | 706 | 46 |  |  |  |  |  |  |
| 4D | 83 | 19 |  |  |  |  |  |  |
| 5A | 1147 | 78 |  |  |  |  |  |  |
| 5B | 1866 | 80 |  |  |  |  |  |  |
| 5D | 208 | 30 |  |  |  |  |  |  |
| 6A | 1260 | 54 |  |  |  |  |  |  |
| 6B | 1291 | 59 |  |  |  |  |  |  |
| 6D | 211 | 25 |  |  |  |  |  |  |
| 7A | 1251 | 111 |  |  |  |  |  |  |
| 7B | 1361 | 63 |  |  |  |  |  |  |
| 7D | 182 | 40 |  |  |  |  |  |  |
|  |  |  |  |  |  |  |  |  |

**B**

|  | **Whole genome** | | **A genome** | | **B genome** | | **D genome** | |
| --- | --- | --- | --- | --- | --- | --- | --- | --- |
|  | **Full SNP set** | **LD pruned r^2^≤0.1** | **Full SNP set** | **LD pruned r^2^≤0.1** | **Full SNP set** | **LD pruned r^2^≤0.1** | **Full SNP set** | **LD pruned r^2^≤0.1** |
| Average distance between SNP (cM) | 0.52 | 9.06 | 0.51 | 8.56 | 0.36 | 7.76 | 1.35 | 13.28 |
| Largest distance between SNP (cM) | 79.53 | 135.67 | 69.26 | 66.58 | 35.14 | 74.10 | 79.53 | 135.67 |
